# Supplementary material for: Efficient Inhibition of Deep Conversion of Partial Oxidation Products in C-H Bonds’ Functionalization Utilizing O2 via Relay Catalysis of Dual Metalloporphyrins on Surface of Hybrid Silica Possessing Capacity for Product Exclusion
Source: Biomimetics (Basel). 2024 Apr 29;9(5):272. doi: 10.3390/biomimetics9050272 (PMC11117990; doi:10.3390/biomimetics9050272)
Supplement: Supplementary file 1 [file biomimetics-09-00272-s001.zip › biomimetics-2912991-supplementary.pdf]

Supplementary material

1. Experimental section

2. Characterization of catalytic materials

3. Experimental data

4. Apparent kinetic study

5. Mechanism study

6. ^{1}H NMR spectra of porphyrins

7. ^{13}C NMR spectra of prophyrins

8. ESI-MS spectra of porphyrins and metalloporphyrins

9. References

1. Experimental section

1.1 Chemicals and materials

Pyrrole (99%), benzaldehyde (99%), 4-chlorobenzaldehyde (97%), 2,3,6-trichlorobenzaldehyde (97%), 2,4,6-trifluorobenzaldehyde (97%), 4-(trifluoromethyl)benzaldehyde (97%), 3,5-bis(trifluoromethyl)benzaldehyde (97%), pentafluorobenzaldehyde (97%), 4-(chloromethyl)benzaldehyde (97%), anhydrous copper(II) acetate (98%), anhydrous zinc(II) acetate (98%), anhydrous cobalt(II) acetate (98%) and anhydrous nickel(II) acetate (98%) utilized in the syntheses of metalloporphyrins were purchased from Shanghai Titan Technology Co., Ltd., Energy Chemical Co., Ltd., shanghai Lingfeng Chemical Reagent Co., Ltd., Shanghai Aladdin Biochemical Technology Co., Ltd., and Shanghai Bide Pharmaceutical Technology Co., Ltd, respectively. Tetraethoxysilane (98%), 3-(aminopropyl)triethoxysilane (98%), tetrabutylammonium fluoride (1 mol/L in THF), and anhydrous ethanol (analytical pure) employed in the preparation of hybrid silica were purchased from Shanghai Titan Technology Co., Ltd., Energy Chemical Co., Ltd., shanghai Lingfeng Chemical Reagent Co., Ltd., and Shanghai Aladdin Biochemical Technology Co., Ltd., respectively. Potassium iodide (99%), anhydrous potassium carbonate (99%) and N,N-dimethylformamide (analytical pure) utilized in the immobilization of metalloporphyrins on hybrid silica were the commodities of Shanghai Titan Technology Co., Ltd. and J&K Scientific Co., Ltd., respectively. Cycloalkanes and aromatics in their highest purities available employed as substrates in catalytic oxidation were the commodities of Aladdin Biochemical Technology Co., Ltd., Sinopharm Chemical Reagent Co., Ltd., Macklin Biochemical Technology Co., Ltd., Shanghai Titan Technology Co., Ltd., and Guangdong Guanghua Sci-Tech Co., Ltd., respectively. All the cycloalkanes were dried over sodium and redistilled before utilization in catalytic oxidation as substrates. Cycloalkanols, cycloalkanones, aromatic alcohols, aromatic ketones and carboxylic acids utilized as standard samples in their highest purities available in GC and HPLC analyses were purchased from Alfa Aesar, TCI, Meryer, Titan and Macklin respectively. Common reagents in the experiments were purchased

from Hangzhou Shuanglin Chemical Reagent Co., Ltd., Sinopharm Chemical Reagent Co., Ltd., Guangdong Guanghua Sci-Tech Co., Ltd., Shanghai Titan Technology Co., Ltd., and Shanghai Lingfeng Chemical Reagent Co., Ltd., respectively and used directly without any purification.

1.2 Characterizations and instruments

The ^{1}H NMR and ^{13}C NMR spectra were collected on a Bruker AVANCE III (500MHz) spectrometer employing tetramethylsilane (TMS) as internal standard and $CDCl_3$ or DMSO-d_6 as solvent. The ESI-MS spectra were recorded on an Agilent 6210 LC/TOF mass spectrometer equipped with electrospray ionization source in the direct injection mode. The GC-MS measurements were conducted on an Agilent 8890-7000D gas chromatography-triple quadrupole mass spectrometer with an electron impact ion source. The FT-IR spectra were measured on a Thermo Nicolet 6700 spectrometer using KBr as background over the frequency range of 4000~400 cm^{-1}. The XPS analyses were conducted on a Kratos AXIS Ultra DLD spectrometer. The SEM images were recorded on a Zeiss Gemini 500 microscope. The TEM images were collected on a Hitachi HT 7700 microscope. The EDS analyses were carried out on an energy dispersive spectrometer coupled with the SEM and TEM analyses. The TG analyses were performed on a PerkinElmer Diamond TG/DTA instrument under air atmosphere from room temperature to 800 °C at a speed of 10 °C/min. The XRD patterns were collected on a PANalytical Empyrean powder diffractometer using Cu Kα radiation (λ = 0.1541 nm) and the 2θ range in the XRD measurement was from 10° to 80° with a step of 0.0167°. The contact angles of cyclohexane, cyclohexanol and cyclohexanone on the surface of catalytic material were measured through the KRÜSS DSA 100S Automatic Contact Angle Measuring Instrument. The EPR analyses were conducted on a JEOL JES-FA200 spectrometer to investigate the free radical species in the catalytic system. And 5,5-dimethyl-1-pyrroline N-Oxide (DMPO) was used as the radical trapping agent. The UV-Vis data were collected on a HITACHI U-3900 spectrometer using cyclohexane as solvent in a quartz cuvette at room temperature. The GC analyses

were carried out employing a TG-5MS capillary column (30 m × 0.32 mm × 0.25 μm) on a Thermo Scientific Trace 1300 instrument. In GC analyses, toluene was utilized as internal standard and acetone was employed to dissolve the samples. Both the temperature of gasification chamber and detector was set as 250 °C, and the initial column temperature was set as 60 °C and kept for 1.0 min. Then the column temperature was raised to 250 °C at the speed of 10 °C/min and was kept at 250 °C for 10.0 min. The split ratio in GC analyses was 1 : 40. The HPLC analyses were conducted employing an Amethyst C18-H chromatography column (150 mm × 4.6 mm× 0.25 μm) on a Thermo Scientific Ultimate 3000 chromatography equipped with an Ultimate 3000 Photodiode Array Detector. In HPLC analyses, benzoic acid was utilized as internal standard and methanol was employed to dissolve the samples.

1.3 Syntheses of porphyrins

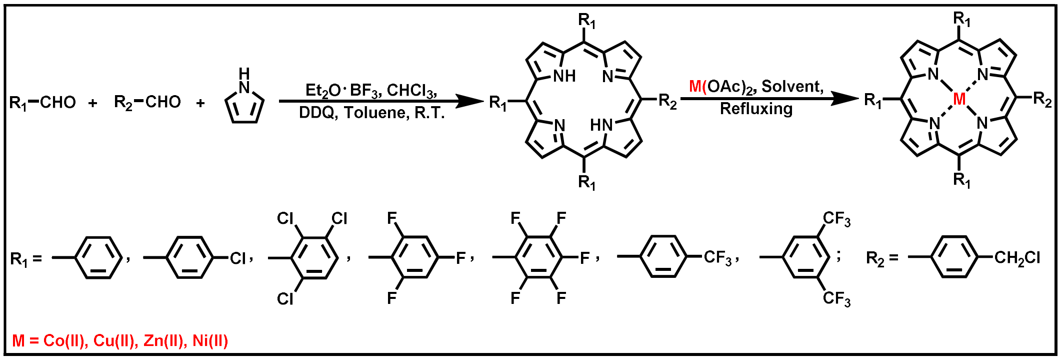

Scheme S1. Schematic syntheses of A_3B-type metalloporphyrins.

The syntheses of A_4-type porphyrins were conducted through the procedure presented in our previous works [9,15,26,33], and the obtained porphyrins were confirmed via ^{1}H NMR and thin-layer chromatography. The A_3B-type porphyrins were synthesized through modifying the procedure above. A typical procedure for the syntheses of A_3B-type porphyrins was illustrated as follow. In a three-neck round-bottom flask (2000 mL), aromatic aldehyde A (37.50 mmol) and aromatic aldehyde B (12.50 mmol) were dissolved in $CHCl_3$ (1500 mL) under the protection of nitrogen , and then redistilled pyrrole (3.35 g, 50.00 mmol) was added dropwise to the obtained

solution with the shelter from light utilizing tinfoil. After stirring at room temperature for 15.0 min in the shelter from light and under the protection of nitrogen, boron trifluoride etherate ($BF_3 \cdot OEt_2$, 48%) (1.48 g, 5.0 mmol) was added to the colorless reaction mixture. After reaction of 30.0 min, 2,3-dicyano-5,6-dichlorobenzoquinone (DDQ) (11.35 g, 45.00 mmol) in toluene (100 mL) was added in about 30.0 min. The resultant reaction mixture was kept stirring at room temperature for 1.0 h in the shelter from light and under the protection of nitrogen. The black solid obtained through evaporation of the solvent was purified for the first time through neutral aluminum oxide column chromatography (200-300 meso) utilizing CH_2Cl_2 as eluent. All the red to black eluent was collected, and the solvent was evaporated. Then the obtained black power was purified for the second time through silica gel column chromatography (200-300 meso) employing cyclohexane and dichloromethane in the volume ratio of 4 : 1 as eluent. The obtained purple powder was dried at 80 °C under reduced pressure for 12.0 h to afford A_3B-type porphyrins. All the obtained porphyrins were characterized and confirmed through ^1H NMR, ^{13}C NMR and ESI-MS.

(1) 5-(4-(Chloromethyl)phenyl)-10,15,20-triphenylporphyrin (TrisP(4-CH_2Cl)PP). Benzaldehyde (3.9795 g, 37.50 mmol), 4-(chloromethyl)benzaldehyde (1.9324 g, 12.50 mmol), cyclohexane and dichloromethane as eluent in the volume ratio of 4 : 1. Purple power 0.4700 g in the yield of 5.67%. ^1H NMR (500 MHz, $CDCl_3$): δ = 8.84-8.83 (m, 8H), 8.22 (d, 8H), 7.79-7.74 (m, 11H), 4.95 (s, 2H), -2.78 (s, 2H). ^{13}C NMR (125 MHz, $CDCl_3$): δ = 142.40, 142.16, 136.97, 134.87, 134.60, 127.78, 126.96, 126.74, 124.43, 123.54, 120.36, 120.28, 46.32. ESI-MS(m/z): 663.2 $[M+H]^+$.

(2) 5-(4-(Chloromethyl)phenyl)-10,15,20-tris(4-chlorophenyl)porphyrin (Tris(4-Cl)P(4-CH_2Cl)PP). 4-Chlorobenzaldehyde (5.2714 g, 37.50 mmol), 4-(chloromethyl)benzaldehyde (1.9324 g, 12.50 mmol), cyclohexane and dichloromethane as eluent in the volume ratio of 4 : 1. Purple power 0.4584 g in the yield of 4.78%. ^1H NMR (500 MHz, $CDCl_3$): δ = 8.85-8.81 (m, 8H), 8.18 (d, 2H), 8.12 (d, 6H), 7.77-7.71 (m, 8H), 4.93 (s, 2H), -2.85 (s, 2H). ^{13}C NMR (125 MHz, $CDCl_3$): δ = 142.06, 140.42, 137.17, 135.52, 134.82, 134.38, 127.05, 124.39, 118.93, 46.25. ESI-MS(m/z): 765.2 $[M+H]^+$.

(3) 5-(4-(Chloromethyl)phenyl)-10,15,20-tris(2,3,6-trichlorophenyl)porphyrin (Tris(2,3,6-triCl)P(4-CH_2Cl)PP). 2,3,6-Trichlorobenzaldehyde (7.8544 g, 37.50 mmol), 4-(chloromethyl)benzaldehyde (1.9324 g, 12.50 mmol), cyclohexane and dichloromethane as eluent in the volume ratio of 4 : 1. Purple power 0.7153 g in the yield of 5.88%. ^{1}H NMR (500 MHz, $CDCl_3$): δ = 8.93 (d, 2H), 8.75-8.71 (m, 6H), 8.23 (d, 2H), 7.81-7.66 (m, 8H), 4.93 (s, 2H), -2.47 (s, 2H). ^{13}C NMR (125 MHz, $CDCl_3$): δ = 141.58, 141.28, 141.01, 137.29, 136.96, 136.91, 134.77, 133.94, 132.06, 131.37, 130.02, 128.14, 127.02, 120.89, 46.22. ESI-MS(m/z): 972.9 $[M+H]^+$.

(4) 5-(4-(Chloromethyl)phenyl)-10,15,20-tris(2,4,6-trifluorophenyl)porphyrin (Tris(2,4,6-triF)P(4-CH_2Cl)PP). 2,4,6-Trifluorobenzaldehyde (6.0037 g, 37.50 mmol), 4-(chloromethyl)benzaldehyde (1.9324 g, 12.50 mmol), cyclohexane and dichloromethane as eluent in the volume ratio of 4 : 1. Purple power 0.6127 g in the yield of 5.94%. ^{1}H NMR (500 MHz, $CDCl_3$): δ = 9.09-8.92 (m, 8H), 8.30 (d, 2H), 7.91 (d, 2H), 7.84-7.80 (m, 6H), 5.16 (s, 2H), -3.04 (s, 2H). ^{13}C NMR (125 MHz, $CDCl_3$): δ = 164.75, 163.72, 162.25, 161.38, 141.64, 134.79, 127.00, 121.16, 105.18, 104.28, 100.34, 46.18. ESI-MS(m/z): 825.2 $[M+H]^+$.

(5) 5-(4-(Chloromethyl)phenyl)-10,15,20-tris(perfluorophenyl)porphyrin (Tris(perF)P(4-CH_2Cl)PP). 2,3,4,5,6-Pentafluorobenzaldehyde (7.3530 g, 37.50 mmol), 4-(chloromethyl)benzaldehyde (1.9324 g, 12.50 mmol), cyclohexane and dichloromethane as eluent in the volume ratio of 4 : 1. Purple power 0.7363 g in the yield of 6.31%. ^{1}H NMR (500 MHz, $CDCl_3$): δ = 8.90 (dt, 8H), 8.21 (d, 2H), 7.81 (d, 2H), 4.95 (s, 2H), -2.85 (s, 2H). ^{13}C NMR (125 MHz, $CDCl_3$): δ = 147.81, 145.32, 141.09, 138.85, 137.71, 136.36, 134.85, 127.18, 122.44, 103.10, 102.01, 46.08. ESI-MS(m/z): 931.1 $[M-H]^-$.

(6) 5-(4-(Chloromethyl)phenyl)-10,15,20-tris(4-(trifluoromethyl)phenyl)-porphyrin (Tris(4-CF_3)P(4-CH_2Cl)PP). 4-(Trifluoromethyl)benzaldehyde (6.5295 g, 37.50 mmol), 4-(chloromethyl)benzaldehyde (1.9324 g, 12.50 mmol), cyclohexane and dichloromethane as eluent in the volume ratio of 4 : 1. Purple power 0.6363 g in the yield of 5.87%. ^{1}H NMR (500 MHz, $CDCl_3$): δ = 8.87-8.76 (m, 8H), 8.29 (d, 6H), 8.17 (d, 2H), 7.99 (d, 6H), 7.74 (d, 2H), 4.90 (s, 2H), -2.82 (s, 2H). ^{13}C NMR (125 MHz,

$CDCl_3$): δ = 145.61, 141.87, 137.31, 134.83, 134.60, 130.46, 130.13, 127.08, 125.89, 123.82, 123.19, 120.36, 118.83, 118.67, 46.19. ESI-MS(m/z): 867.2 $[M+H]^+$.

(7) 5-(4-(Chloromethyl)phenyl)-10,15,20-tris(3,5-bis(trifluoromethyl)phenyl)-porphyrin (Tris(3,5-diCF$_3$)P(4-CH$_2$Cl)PP). 3,5-Bis(trifluoromethyl)benzaldehyde (9.0795 g, 37.50 mmol), 4-(chloromethyl)benzaldehyde (1.9324 g, 12.50 mmol), cyclohexane and dichloromethane as eluent in the volume ratio of 4 : 1. Purple power 0.7070 g in the yield of 5.28%. ^{1}H NMR (500 MHz, $CDCl_3$): δ = 8.98-8.71(m, 14H), 8.39 (s, 3H), 8.23 (d, 2H), 7.83 (d, 2H), 4.96 (s, 2H), -2.85 (s, 2H). ^{13}C NMR (125 MHz, $CDCl_3$): δ = 143.84, 141.40, 137.67, 134.86, 133.74, 130.74, 130.40, 127.21, 124.86, 124.41, 123.52, 122.15, 121.54, 117.16, 116.81, 46.09. ESI-MS(m/z): 1071.2 $[M+H]^+$.

1.4 Syntheses of porphyrin cobalts(II)

The A_3B-type porphyrin cobalts(II) in this work were synthesized through the procedure presented in our previous works with some modifications [9,15,26,33]. And the obtained porphyrin cobalts(II) were confirmed via ESI-MS. A typical procedure for the syntheses of A_3B-type porphyrin cobalts(II) was illustrated as follow. In a three-neck round-bottom flask (100 mL), A_3B-type porphyrin (0.40 mmol) was dissolved in anhydrous methanol (40 mL) under the protection of nitrogen. And then the obtained reaction mixture was heated to reflux with stirring. Under refluxing, anhydrous cobalt (II) acetate (0.7081 g, 4.00 mmol) was added with the protection of nitrogen. After another refluxing for 48.0 h under the protection of nitrogen, the reaction mixture was cooled to room temperature, and the solvent was evaporated under reduced pressure. The obtained solid was purified through silica gel column chromatography (200-300 meso) employing cyclohexane and dichloromethane in the volume ratio of 4 : 1 as eluent. The obtained pink powder was dried at 80 °C under reduced pressure for 8.0 h to afford A_3B-type porphyrin cobalts(II). All the obtained porphyrin cobalts(II) were characterized and confirmed through ESI-MS.

(1) 5-(4-(Chloromethyl)phenyl)-10,15,20-triphenylporphyrin cobalt(II) (TrisP(4-CH$_2$Cl)PPCo). 5-(4-(Chloromethyl)phenyl)-10,15,20-triphenylporphyrin (0.2653 g,

0.40 mmol), anhydrous cobalt (II) acetate (0.7081 g, 4.00 mmol), cyclohexane and dichloromethane as eluent in the volume ratio of 4 : 1. Pink power 0.1731 g in the yield of 60.1%. ESI-MS(m/z): 719.1 $[M]^+$.

(2) 5-(4-(Chloromethyl)phenyl)-10,15,20-tris(4-chlorophenyl)porphyrin cobalt(II) (Tris(4-Cl)P(4-CH$_2$Cl)PPCo). 5-(4-(Chloromethyl)phenyl)-10,15,20-tri(4-chlorophenyl)porphyrin (0.3066 g, 0.40 mmol), anhydrous cobalt (II) acetate (0.7081 g, 4.00 mmol), cyclohexane and dichloromethane as eluent in the volume ratio of 4 : 1. Pink power 0.1835 g in the yield of 55.7%. ESI-MS(m/z): 823.1 $[M]^+$.

(3) 5-(4-(Chloromethyl)phenyl)-10,15,20-tris(2,3,6-trichlorophenyl)porphyrin cobalt(II) (Tris(2,3,6-triCl)P(4-CH$_2$Cl)PPCo). 5-(4-(Chloromethyl)phenyl)-10,15,20-tris(2,3,6-trichlorophenyl)porphyrin (0.3893 g, 0.40 mmol), anhydrous cobalt (II) acetate (0.7081 g, 4.00 mmol), cyclohexane and dichloromethane as eluent in the volume ratio of 4 : 1. Pink power 0.2567 g in the yield of 62.3%. ESI-MS(m/z): 1028.8 $[M]^+$.

(4) 5-(4-(Chloromethyl)phenyl)-10,15,20-tris(2,4,6-trifluorophenyl)porphyrin cobalt(II) (Tris(2,4,6-triF)P(4-CH$_2$Cl)PPCo). 5-(4-(Chloromethyl)phenyl)-10,15,20-tris(2,4,6-trifluorophenyl)porphyrin (0.3301 g, 0.40 mmol), anhydrous cobalt (II) acetate (0.7081 g, 4.00 mmol), cyclohexane and dichloromethane as eluent in the volume ratio of 4 : 1. Pink power 0.2314 g in the yield of 65.6%. ESI-MS(m/z): 881.1 $[M]^+$.

(5) 5-(4-(Chloromethyl)phenyl)-10,15,20-tris(perfluorophenyl)porphyrin cobalt(II) (Tris(perF)P(4-CH$_2$Cl)PPCo). 5-(4-(Chloromethyl)phenyl)-10,15,20-tris(perfluorophenyl)porphyrin (0.3732 g, 0.40 mmol), anhydrous cobalt (II) acetate (0.7081 g, 4.00 mmol), cyclohexane and dichloromethane as eluent in the volume ratio of 4 : 1. Pink power 0.2249 g in the yield of 56.8%. ESI-MS(m/z): 989.0 $[M]^+$.

(6) 5-(4-(Chloromethyl)phenyl)-10,15,20-tris(4-(trifluoromethyl)phenyl)-porphyrin cobalt(II) (Tris(4-CF$_3$)P(4-CH$_2$Cl)PPCo). 5-(4-(Chloromethyl)phenyl)-10,15,20-tris(4-(trifluoromethyl)phenyl)porphyrin (0.3469 g, 0.40 mmol), anhydrous cobalt (II) acetate (0.7081 g, 4.00 mmol), cyclohexane and dichloromethane as eluent in the volume ratio of 4 : 1. Pink power 0.2222 g in the yield of 60.1%. ESI-MS(m/z):

923.1 $[M]^+$.

(7) 5-(4-(Chloromethyl)phenyl)-10,15,20-tris(3,5-bis(trifluoromethyl)phenyl)-porphyrin cobalt(II) (Tris(3,5-diCF$_3$)P(4-CH$_2$Cl)PPCo). 5-(4-(Chloromethyl)phenyl)-10,15,20-tris(3,5-bis(trifluoromethyl)phenyl)porphyrin (0.4285 g, 0.40 mmol), anhydrous cobalt (II) acetate (0.7081 g, 4.00 mmol), cyclohexane and dichloromethane as eluent in the volume ratio of 4 : 1. Pink power 0.2658 g in the yield of 58.9%. ESI-MS(m/z): 1127.1 $[M]^+$.

1.5 Syntheses of porphyrin coppers(II)

The A_3B-type porphyrin coppers(II) in this work were synthesized through the procedure presented in our previous works with some modifications [9,15,26,33]. And the obtained porphyrin coppers(II) were confirmed via ESI-MS. A typical procedure for the syntheses of A_3B-type porphyrin coppers(II) was illustrated as follow. In a three-neck round-bottom flask (250 mL), A_3B-type porphyrin (0.40 mmol) was dissolved in chloroform (120 mL) under the protection of nitrogen. And then the obtained reaction mixture was heated to reflux with stirring. Under refluxing, anhydrous copper (II) acetate (0.7265 g, 4.00 mmol) was added with the protection of nitrogen. After another refluxing for 12.0 h under the protection of nitrogen, the reaction mixture was cooled to room temperature, and the solvent was evaporated under reduced pressure. The obtained solid was purified through silica gel column chromatography (200-300 meso) employing cyclohexane and dichloromethane in the volume ratio of 4 : 1 as eluent. The obtained red powder was dried at 80 °C under reduced pressure for 8.0 h to afford A_3B-type porphyrin coppers(II). All the obtained porphyrin coppers(II) were characterized and confirmed through ESI-MS.

(1) 5-(4-(Chloromethyl)phenyl)-10,15,20-triphenylporphyrin copper(II) (TrisP(4-CH$_2$Cl)PPCu). 5-(4-(Chloromethyl)phenyl)-10,15,20-triphenylporphyrin (0.2653 g, 0.40 mmol), anhydrous copper (II) acetate (0.7265 g, 4.00 mmol), cyclohexane and dichloromethane as eluent in the volume ratio of 4 : 1. Red power 0.2191 g in the yield of 75.6%. ESI-MS(m/z): 723.2 $[M]^+$.

(2) 5-(4-(Chloromethyl)phenyl)-10,15,20-tris(4-chlorophenyl)porphyrin copper(II) (Tris(4-Cl)P(4-CH_2Cl)PPCu). 5-(4-(Chloromethyl)phenyl)-10,15,20-tri(4-chlorophenyl)porphyrin (0.3066 g, 0.40 mmol), anhydrous copper (II) acetate (0.7265 g, 4.00 mmol), cyclohexane and dichloromethane as eluent in the volume ratio of 4 : 1. Red power 0.2577 g in the yield of 77.8%. ESI-MS(m/z): 825.0 $[M]^+$.

(3) 5-(4-(Chloromethyl)phenyl)-10,15,20-tris(2,3,6-trichlorophenyl)porphyrin copper(II) (Tris(2,3,6-triCl)P(4-CH_2Cl)PPCu). 5-(4-(Chloromethyl)phenyl)-10,15,20-tris(2,3,6-trichlorophenyl)porphyrin (0.3893 g, 0.40 mmol), anhydrous copper (II) acetate (0.7265 g, 4.00 mmol), cyclohexane and dichloromethane as eluent in the volume ratio of 4 : 1. Red power 0.3373 g in the yield of 81.5%. ESI-MS(m/z): 1032.8 $[M]^+$.

(4) 5-(4-(Chloromethyl)phenyl)-10,15,20-tris(2,4,6-trifluorophenyl)porphyrin copper(II) (Tris(2,4,6-triF)P(4-CH_2Cl)PPCu). 5-(4-(Chloromethyl)phenyl)-10,15,20-tris(2,4,6-trifluorophenyl)porphyrin (0.3301 g, 0.40 mmol), anhydrous copper (II) acetate (0.7265 g, 4.00 mmol), cyclohexane and dichloromethane as eluent in the volume ratio of 4 : 1. Red power 0.2667 g in the yield of 75.2%. ESI-MS(m/z): 885.1 $[M]^+$.

(5) 5-(4-(Chloromethyl)phenyl)-10,15,20-tris(perfluorophenyl)porphyrin copper(II) (Tris(perF)P(4-CH_2Cl)PPCu). 5-(4-(Chloromethyl)phenyl)-10,15,20-tris(perfluorophenyl)porphyrin (0.3732 g, 0.40 mmol), anhydrous copper (II) acetate (0.7265 g, 4.00 mmol), cyclohexane and dichloromethane as eluent in the volume ratio of 4 : 1. Red power 0.3131 g in the yield of 78.7%. ESI-MS(m/z): 995.0 $[M]^+$.

(6) 5-(4-(Chloromethyl)phenyl)-10,15,20-tris(4-(trifluoromethyl)phenyl)-porphyrin copper(II) (Tris(4-CF_3)P(4-CH_2Cl)PPCu). 5-(4-(Chloromethyl)phenyl)-10,15,20-tris(4-(trifluoromethyl)phenyl)porphyrin (0.3469 g, 0.40 mmol), anhydrous copper (II) acetate (0.7265 g, 4.00 mmol), cyclohexane and dichloromethane as eluent in the volume ratio of 4 : 1. Red power 0.2809 g in the yield of 75.6%. ESI-MS(m/z): 927.1 $[M]^+$.

(7) 5-(4-(Chloromethyl)phenyl)-10,15,20-tris(3,5-bis(trifluoromethyl)phenyl)-porphyrin copper(II) (Tris(3,5-diCF_3)P(4-CH_2Cl)PPCu). 5-(4-

(Chloromethyl)phenyl)-10,15,20-tris(3,5-bis(trifluoromethyl)phenyl)porphyrin (0.4285 g, 0.40 mmol), anhydrous copper (II) acetate (0.7265 g, 4.00 mmol), cyclohexane and dichloromethane as eluent in the volume ratio of 4 : 1. Red power 0.3276 g in the yield of 72.3%. ESI-MS(m/z): 1131.1 $[M]^+$.

1.6 Syntheses of porphyrin zincs(II)

The A_3B-type porphyrin zincs(II) in this work were synthesized through the procedure presented in our previous works with some modifications [9,15,26,33]. And the obtained porphyrin zincs(II) were confirmed via ESI-MS. A typical procedure for the syntheses of A_3B-type porphyrin zincs(II) was illustrated as follow. In a three-neck round-bottom flask (250 mL), A_3B-type porphyrin (0.40 mmol) and anhydrous zinc acetate (0.7339 g, 4.00 mmol) were dissolved in mixed solvent of dichloromethane (120 mL) and methanol (10 mL) under the protection of nitrogen. And then the obtained reaction mixture was stirred at room temperature for 6.0 h under the protection of nitrogen. When the reaction was finished, the solvent was evaporated under reduced pressure. The obtained solid was purified through silica gel column chromatography (200-300 meso) employing cyclohexane and dichloromethane in the volume ratio of 4 : 1 as eluent. The obtained purplish red powder was dried at 80 °C under reduced pressure for 8.0 h to afford A_3B-type porphyrin zincs(II). All the obtained porphyrin zincs(II) were characterized and confirmed through ESI-MS.

(1) 5-(4-(Chloromethyl)phenyl)-10,15,20-triphenylporphyrin zinc(II) (TrisP(4-CH_2Cl)PPZn). 5-(4-(Chloromethyl)phenyl)-10,15,20-triphenylporphyrin (0.2653 g, 0.40 mmol), anhydrous zinc (II) acetate (0.7339 g, 4.00 mmol), cyclohexane and dichloromethane as eluent in the volume ratio of 4 : 1. Purplish red power 0.2072 g in the yield of 71.3%. ESI-MS(m/z): 724.2 $[M]^+$.

(2) 5-(4-(Chloromethyl)phenyl)-10,15,20-tris(perfluorophenyl)porphyrin zinc(II) (Tris(perF)P(4-CH_2Cl)PPZn). 5-(4-(Chloromethyl)phenyl)-10,15,20-tris(perfluorophenyl)porphyrin (0.3732 g, 0.40 mmol), anhydrous zinc (II) acetate (0.7339 g, 4.00 mmol), cyclohexane and dichloromethane as eluent in the volume ratio

of 4 : 1. Purplish red power 0.2797 g in the yield of 70.2%. ESI-MS(m/z): 994.0 $[M]^+$.

1.7 Preparation of hybrid silica (Si@NH_2)

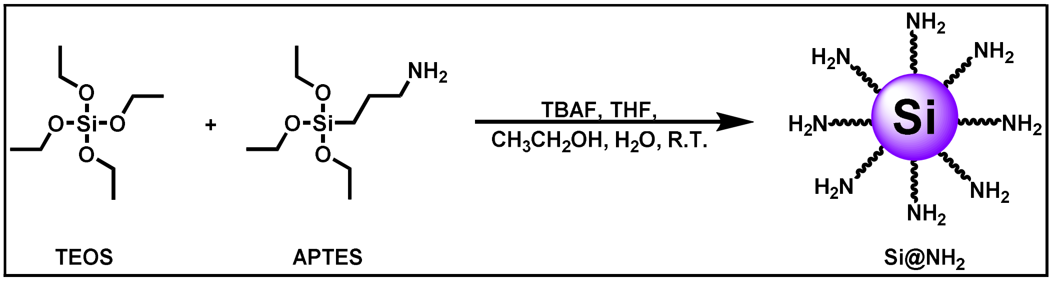

For preparation of hybrid silica (Si@NH_2), two solutions (A and B) were prepared. In a beaker (50 mL), tetraethoxysilane (10.4165 g, 50.0 mmol) and 3-(aminopropyl)triethoxysilane (2.2137 g, 10.0 mmol) were dissolved in anhydrous ethanol (30 mL) as solution A. Tetrabutylammonium fluoride (3.0 mL, 1.0 mol/L in THF) and distilled water (3.6040 g, 200.0 mmol) were dissolved in anhydrous ethanol (20 mL) as solution B. The solution A and solution B were mixed in a reaction bottle and the obtained mixture was shaken at room temperature for 30 s. A white gel would be formed, and the obtained white gel was aged at room temperature for 6.0 days. The collected solid was washed successively by ethanol (50 mL) and acetone (50 mL), and dried at 60 °C under reduced pressure for 6.0 h to afford hybrid silica (Si@NH_2) as white power (2.3675 g).

1.8 Apparent kinetics study

In this work, apparent kinetics studies were utilized to investigate the catalytic performance of the developed immobilized dual metalloporphyrins in partial oxidation of C-H bonds with O_2, in which cyclohexane was utilized as model substrate possessing C-H bonds, and optimized catalytic materials Si@Porp. Co and Si@Porp. Co&Cu were utilized as representative catalysts. All the apparent kinetics experiments were conducted in a high-pressure reactor made from stainless steel, equipped with an inner lining made from polytetrafluoroethylene. In the autoxidation without catalyst,

cyclohexane (16.8320 g, 200 mmol) was heated to target temperature (130 °C, 135 °C and 140 °C respectively) with stirring, and O_2 (1.0 MPa) was introduced into the reactor at a state of continuous supply. The reaction was stopped at the reaction time of 1.0 h, 2.0 h, 3.0 h, 4.0 h, and 5.0 h respectively through reducing the reaction temperature to room temperature quickly in an ice-water bath. When the high-pressure reactor was opened, triphenylphosphine (1.3115 g, 5.00 mmol) was added quickly with another stirring of 30.0 min to transform the formed hydroperoxides. The reaction mixture was dissolved in acetone to 100 mL exactly to determine the substrate conversions in autoxidation through GC and HPLC analyses as illustrated in 2.2. In the catalytic oxidation of cyclohexane, catalyst (0.0320 g, 0.16 g/mol) was dispersed in cyclohexane (16.8320 g, 200 mmol) with stirring, and the reaction mixture was heated to target temperature (110 °C, 115 °C and 120 °C respectively) with stirring. Then O_2 (1.0 MPa) was introduced into the reactor at a state of continuous supply. The reaction was stopped at the reaction time of 4.0 h, 5.0 h, 6.0 h, 7.0 h, and 8.0 h respectively through reducing the reaction temperature to room temperature quickly in an ice-water bath. When the high-pressure reactor was opened, triphenylphosphine (1.3115 g, 5.00 mmol) was added quickly with another stirring of 30.0 min to transform the formed hydroperoxides. The reaction mixture was dissolved in acetone to 100 mL exactly to determine the substrate conversions in catalytic oxidation through GC and HPLC analyses as illustrated in manuscript 2.2.

1.9 Free radical capture experiment

To capture the free radical species in partial oxidation of C-H bonds with O_2, cyclohexane was utilized as model substrate containing C-H bonds, optimized Si@Porp. Co&Cu was utilized as catalyst, and bromochloroform, *tert*-butyl bromide and diphenylamine were utilized as capture reagents in this work to conduct the free radical capture experiments. In a high-pressure reactor made from stainless steel, equipped with an inner lining made from polytetrafluoroethylene (100 mL), optimized Si@Porp. Co&Cu (0.0320 g, 0.16 mg/mmol) was dispersed in cyclohexane (16.8320 g, 200

mmol), and capture reagent (bromochloroform, *tert*-butyl bromide or diphenylamine, 5.00 mmol) was added under the condition of stirring. Then in an oil bath, the temperature of the reaction mixture was raised to 120 °C, and O_2 flowed into the sealed high-pressure reactor to raise the reaction pressure to 1.0 MPa. After reaction at 120 °C and 1.0 MPa for 8.0 h, the reaction mixture was cooled quickly to room temperature in an ice-water bath. When the high-pressure reactor was opened, triphenylphosphine (1.3115 g, 5.00 mmol) was added quickly with another stirring of 30.0 min to transform the formed hydroperoxides. At last, the reaction mixture was dissolved in acetone to 100 mL exactly to conduct GC-MS, GC and HPLC analyses. Through quantitative and qualitative analyses, both of the suppressed substrate conversion and the detailed information of captured free radical species were obtained.

1.10 EPR measurement

To further explore the free radical species in partial oxidation of C-H bonds with O_2, EPR (Electron paramagnetic resonance) measurement was carried out with cyclohexane as model substrate containing C-H bonds, optimized Si@Porp. Co&Cu as catalyst, and 5,5-dimethyl-1-pyrroline N-Oxide (DMPO) as radical trapping agent. In a high-pressure reactor made from stainless steel, equipped with an inner lining made from polytetrafluoroethylene (100 mL), Si@Porp. Co&Cu (0.0320 g, 0.16 mg/mmol) was dispersed in cyclohexane (16.8320 g, 200 mmol) under the condition of stirring. Then in an oil bath, the temperature of the reaction mixture was raised to 120 °C, and O_2 flowed into the sealed high-pressure reactor to raise the reaction pressure to 1.0 MPa. After reaction at 120 °C and 1.0 MPa for 6.0 h, 5,5-dimethyl-1-pyrroline N-Oxide (DMPO) (0.0011 g, 0.01 mmol) was added to 1.0 mL of the reaction mixture to trap the free radical species for EPR measurements. The electron paramagnetic resonance (EPR) measurement was conducted on a JEOL JES-FA200 spectrometer under the microwave frequency of 9.26 GHz at room temperature.

2. Characterization of catalytic materials

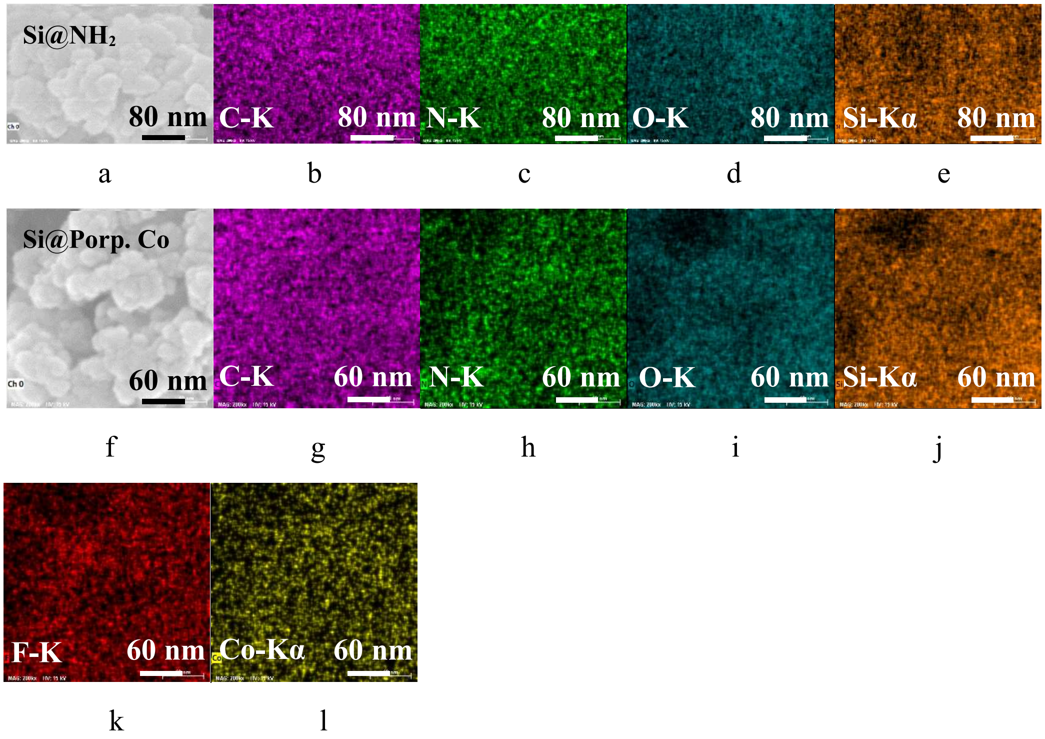

Figure S1. EDS elemental mapping analyses of hybrid silica (Si@NH_2) (a, b, c, d, e), Si@Porp. Co (f, g, h, i, j, k, l) coupled with SEM.

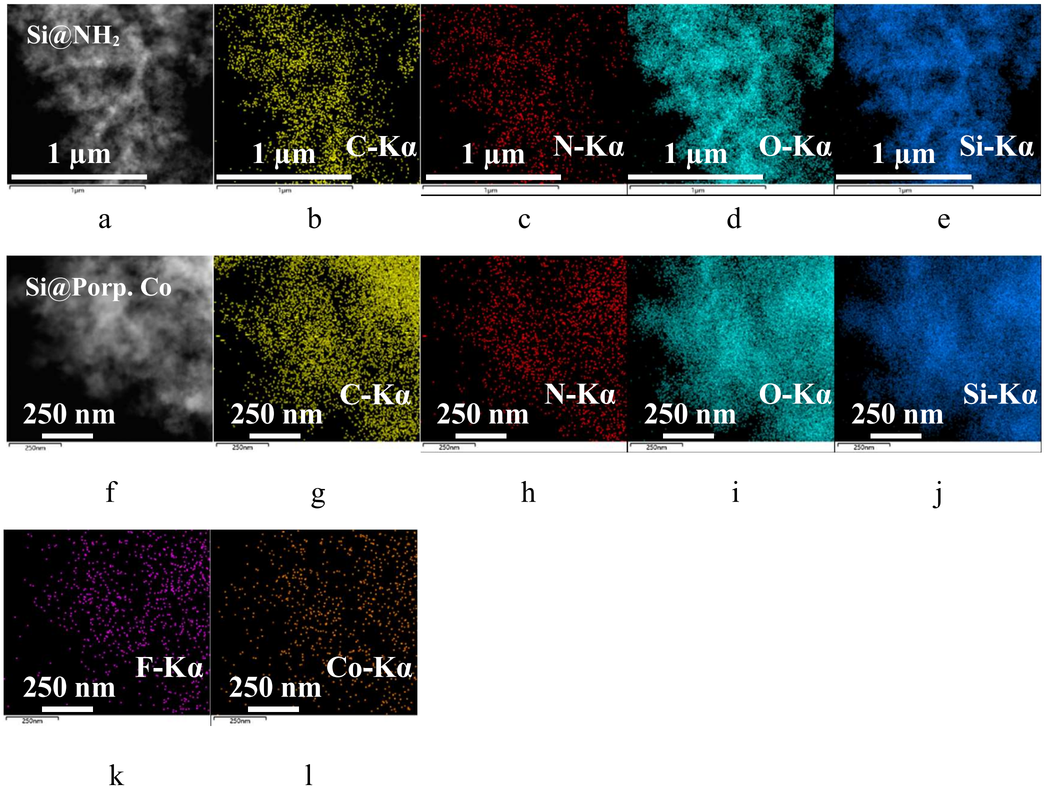

Figure S2. EDS elemental mapping analyses of hybrid silica (Si@NH_2) (a, b, c, d, e), Si@Porp. Co (f, g, h, i, j, k, l) coupled with TEM.

3. Experimental data

Table S1. Effect of ratio between metalloporphyrin moles and Si@NH_2 mass in immobilization on the catalytic performance of Si@Porp. Co.[a]

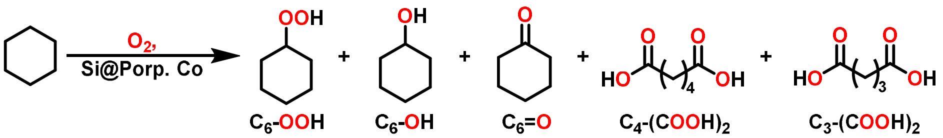

Entry	Ratio (mmol/g)	Conversion (%)	Selectivity (%)					
			C_6-OOH	C_6-OH	C_6=O	C_4-$(COOH)_2$	C_3-$(COOH)_2$	Total[b]
1	0.08	2.22	39.9	25.6	26.1	6.9	1.5	91.6
2	0.10	2.40	40.2	23.3	26.7	5.7	4.1	90.2
3	0.12	2.56	41.5	22.1	27.4	6.6	2.4	91.0
4	0.14	3.44	28.1	30.1	29.4	8.3	4.1	87.6
**5**	**0.16**	**4.47**	**29.3**	**27.0**	**29.4**	**10.0**	**4.3**	**85.7**
6	0.18	4.41	28.5	27.6	30.3	10.1	3.5	86.4
7	0.20	4.46	27.4	27.4	29.3	12.3	3.6	84.1

[a] Reaction conditions: Substrate (Cyclohexane, 16.8320 g, 200 mmol), O_2 (1.0 MPa in constant supply), catalyst Si@Porp. Co (20 mg, 0.10 mg/mmol, catalyst/substrate), stirring at speed of 600 rpm and 120 °C for 8.0 h.

[b] Total selectivity of partial oxidation products (Hydroperoxide, alcohol and ketone).

Table S2. Effect of ratio between metalloporphyrin moles and Si@NH_2 mass in immobilization on the catalytic performance of Si@Porp. Co&Cu.[a]

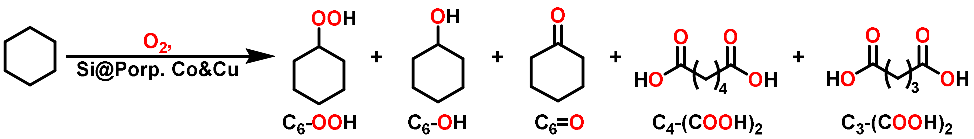

Entry	Ratio	Conversion	Selectivity (%)

	(mmol/g)	(%)	C_6-OOH	C_6-OH	C_6=O	C_4-$(COOH)_2$	C_3-$(COOH)_2$	Total[b]
1	0.08	2.41	36.5	35.6	24.1	3.0	0.8	96.2
2	0.10	2.68	43.3	25.7	21.1	6.9	3.0	90.1
3	0.12	2.70	34.4	30.3	24.5	7.8	3.0	89.2
4	0.14	3.46	26.1	32.9	24.0	12.7	4.3	83.0
**5**	**0.16**	**4.89**	**15.5**	**38.9**	**34.6**	**7.7**	**3.3**	**89.0**
6	0.18	4.92	14.1	39.0	33.4	10.0	3.5	86.5
7	0.20	4.88	20.7	30.4	35.4	9.3	4.2	86.5

[a] Reaction conditions: Substrate (Cyclohexane, 16.8320 g, 200 mmol), O_2 (1.0 MPa in constant supply), catalyst Si@Porp. Co&Cu (20 mg, 0.10 mg/mmol, catalyst/substrate), stirring at speed of 600 rpm and 120 °C for 8.0 h.

[b] Total selectivity of partial oxidation products (Hydroperoxide, alcohol and ketone).

Table S3. Effect of ratio between Co and Cu in Si@Porp. Co&Cu on the catalytic performance of Si@Porp. Co&Cu.[a]

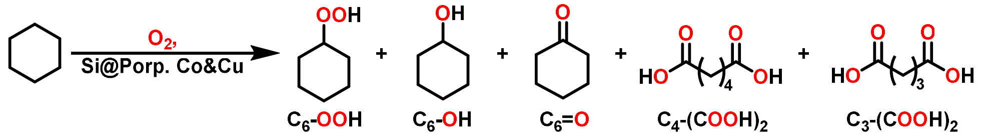

Entry	Ratio (Co : Cu)	Conversion (%)	Selectivity (%)					
			C_6-OOH	C_6-OH	C_6=O	C_4-$(COOH)_2$	C_3-$(COOH)_2$	Total[b]
**1**	**1 : 2**	**5.03**	**17.7**	**33.6**	**38.0**	**8.5**	**2.2**	**89.3**
2	1 : 1	4.89	15.5	38.9	34.6	7.7	3.3	89.0
3	2 : 1	4.67	14.3	39.4	37.7	6.5	2.1	91.4

[a] Reaction conditions: Substrate (Cyclohexane, 16.8320 g, 200 mmol), O_2 (1.0 MPa in constant supply), catalyst Si@Porp. Co&Cu (20 mg, 0.10 mg/mmol, catalyst/substrate), stirring at speed of 600 rpm and 120 °C for 8.0 h. In immobilization reaction, the ratio of metalloporphyrin to Si@NH_2 was 0.16 mmol/g.

[b] Total selectivity of partial oxidation products (Hydroperoxide, alcohol and ketone).

Table S4. Effect of Si@Porp. Co amount on partial oxidation of cyclohexane.[a]

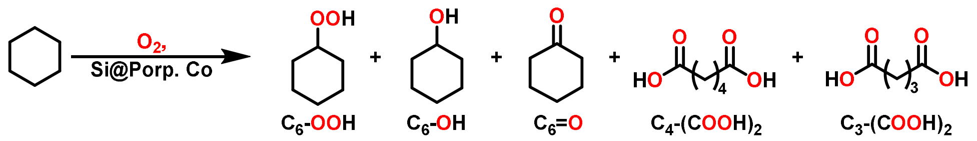

Entry	Catalyst amount (%, g/mol)	Conversion (%)	Selectivity (%)					
			C_6-OOH	C_6-OH	C_6=O	C_4-$(COOH)_2$	C_3-$(COOH)_2$	Total[b]
1	0.10	4.47	31.1	25.4	30.1	9.8	3.6	86.6
2	0.12	4.58	27.1	27.5	30.2	10.1	5.1	84.8
3	0.14	4.76	23.6	26.3	30.6	12.4	7.1	80.5
4	0.16	4.93	25.4	30.3	27.8	11.3	5.2	83.5
**5**	**0.18**	**5.05**	**32.6**	**30.0**	**27.2**	**8.6**	**1.6**	**89.8**
6	0.20	4.44	25.6	31.8	33.4	7.5	1.7	90.8
7	0.22	4.21	32.9	30.8	27.3	6.6	2.4	91.0

[a] Reaction conditions: Substrate (Cyclohexane, 16.8320 g, 200 mmol), O_2 (1.0 MPa in constant supply), catalyst (Si@Porp. Co), stirring at speed of 600 rpm and 120 °C for 8.0 h.

[b] Total selectivity of partial oxidation products (Hydroperoxide, alcohol and ketone).

Table S5. Effect of Si@Porp. Co&Zn amount on partial oxidation of cyclohexane.[a]

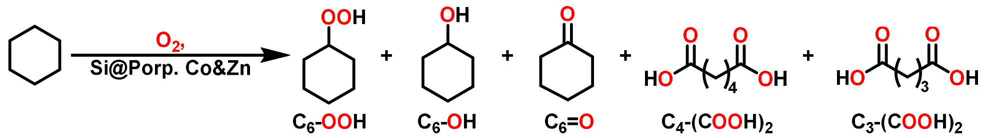

Entry	Catalyst amount (%, g/mol)	Conversion (%)	Selectivity (%)					
			C_6-OOH	C_6-OH	C_6=O	C_4-$(COOH)_2$	C_3-$(COOH)_2$	Total[b]
1	0.10	4.83	42.1	16.9	25.1	12.6	3.3	84.1
2	0.12	4.97	26.5	33.8	22.7	13.7	3.3	83.0
3	0.14	5.07	26.5	32.7	23.1	14.5	3.2	82.3
4	0.16	5.11	35.3	31.7	22.9	8.4	1.7	89.9
**5**	**0.18**	**5.20**	**37.5**	**31.4**	**21.5**	**7.9**	**1.7**	**90.4**

6	0.20	5.19	28.5	36.4	25.2	6.4	3.5	90.1
7	0.22	5.03	16.0	41.6	34.5	5.7	2.2	92.1

[a] Reaction conditions: Substrate (Cyclohexane, 16.8320 g, 200 mmol), O_2 (1.0 MPa in constant supply), catalyst (Si@Porp. Co&Zn), stirring at speed of 600 rpm and 120 °C for 8.0 h.

[b] Total selectivity of partial oxidation products (Hydroperoxide, alcohol and ketone).

Table S6. Effect of pressure on partial oxidation of cyclohexane catalyzed by Si@Porp. Co.[a]

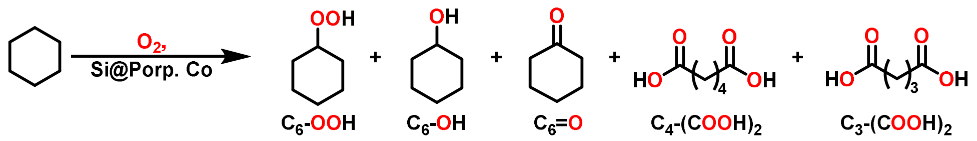

Entry	Pressure (MPa)	Conversion (%)	Selectivity (%)					
			C6-OOH	C6-OH	C6=O	C4-(COOH)2	C3-(COOH)2	Total[b]
1	0.4	2.86	11.1	45.2	40.3	3.4	N. D.[c]	96.6
2	0.6	3.31	10.8	45.2	38.8	3.9	1.3	94.8
3	0.8	4.56	23.8	33.3	32.6	6.6	3.7	89.7
**4**	**1.0**	**5.05**	**32.6**	**30.0**	**27.2**	**8.6**	**1.6**	**89.8**
5	1.2	4.88	24.9	30.2	33.8	9.0	2.1	88.9

[a] Reaction conditions: Substrate (Cyclohexane, 16.8320 g, 200 mmol), O_2 in constant supply, catalyst (Si@Porp. Co, 36 mg, 0.18 mg/mmol), stirring at speed of 600 rpm and 120 °C for 8.0 h.

[b] Total selectivity of partial oxidation products (Hydroperoxide, alcohol and ketone).

[c] No detected.

Table S7. Effect of pressure on partial oxidation of cyclohexane catalyzed by Si@Porp. Co&Zn.[a]

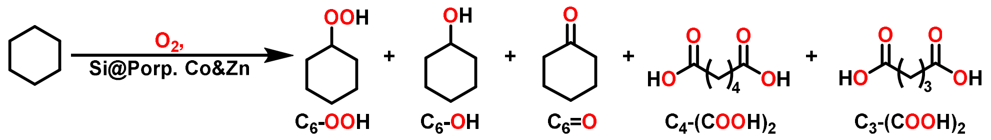

Entry	Pressure (MPa)	Conversion (%)	Selectivity (%)					
			C_6-OOH	C_6-OH	C_6=O	C_4-$(COOH)_2$	C_3-$(COOH)_2$	Total[b]
1	0.4	1.80	39.1	30.7	23.4	5.9	0.9	93.2
2	0.6	2.94	30.3	28.0	36.5	4.6	0.6	94.8
3	0.8	4.39	20.6	43.5	32.3	3.0	0.6	96.4
**4**	**1.0**	**5.20**	**37.5**	**31.4**	**21.5**	**7.9**	**1.7**	**90.4**
5	1.2	4.76	21.2	35.8	31.5	7.1	4.4	88.5

[a] Reaction conditions: Substrate (Cyclohexane, 16.8320 g, 200 mmol), O_2 in constant supply, catalyst (Si@Porp. Co&Zn, 36 mg, 0.18 mg/mmol), stirring at speed of 600 rpm and 120 °C for 8.0 h.

[b] Total selectivity of partial oxidation products (Hydroperoxide, alcohol and ketone).

Table S8. Effect of reaction time on partial oxidation of cyclohexane catalyzed by Si@Porp. Co.[a]

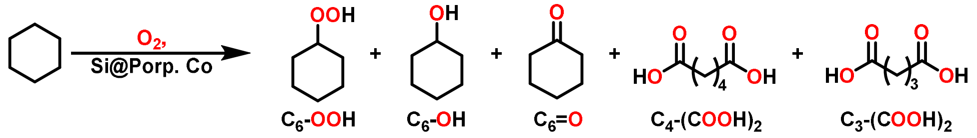

Entry	Reaction time (h)	Conversion (%)	Selectivity (%)					
			C_6-OOH	C_6-OH	C_6=O	C_4-$(COOH)_2$	C_3-$(COOH)_2$	Total[b]
1	4.0	1.56	47.6	11.7	38.2	1.7	0.8	97.5
2	6.0	3.52	15.6	36.7	36.2	7.8	3.7	88.5
**3**	**8.0**	**5.05**	**32.6**	**30.0**	**27.2**	**8.6**	**1.6**	**89.8**
4	10.0	5.09	15.1	30.9	39.6	10.4	4.0	85.6
5	12.0	5.22	12.2	25.3	42.8	15.7	4.0	80.3

[a] Reaction conditions: Substrate (Cyclohexane, 16.8320 g, 200 mmol), O_2 (1.0 MPa in constant supply), catalyst (Si@Porp. Co, 36 mg, 0.18 mg/mmol), stirring at speed of 600 rpm and 120 °C.

[b] Total selectivity of partial oxidation products (Hydroperoxide, alcohol and ketone).

Table S9. Effect of reaction time on partial oxidation of cyclohexane catalyzed by

Si@Porp. Co&Zn.[a]

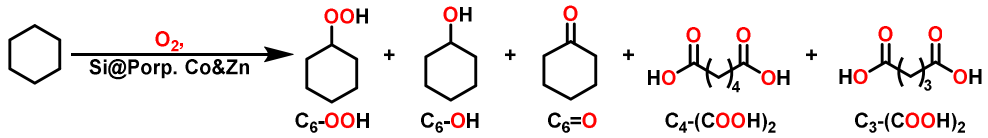

Entry	Reaction time (h)	Conversion (%)	Selectivity (%)					
			C_6-OOH	C_6-OH	C_6=O	C_4-(COOH)$_2$	C_3-(COOH)$_2$	Total[b]
1	4.0	2.03	43.1	26.1	29.0	1.6	0.2	98.2
2	6.0	3.23	20.8	29.5	45.9	3.0	0.8	96.2
**3**	**8.0**	**5.20**	**37.5**	**31.4**	**21.5**	**7.9**	**1.7**	**90.4**
4	10.0	5.29	19.0	30.5	34.8	11.8	3.9	84.3
5	12.0	5.45	22.2	21.7	38.7	12.7	4.7	82.6

[a] Reaction conditions: Substrate (Cyclohexane, 16.8320 g, 200 mmol), O_2 (1.0 MPa in constant supply), catalyst (Si@Porp. Co&Zn, 36 mg, 0.18 mg/mmol), stirring at speed of 600 rpm and 120 °C.

[b] Total selectivity of partial oxidation products (Hydroperoxide, alcohol and ketone).

4. Apparent kinetic study

Table S10. Kinetic data in oxidation of cyclohexane without catalyst.[a]

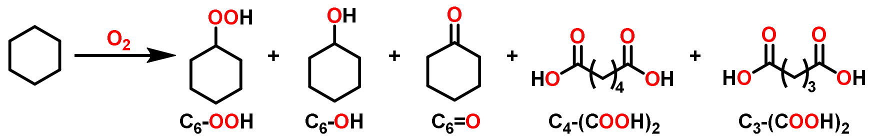

Entry	Temperature (°C)	Reaction time (h)	Conversion (%)	Selectivity (%)					
				C_6-OOH	C_6-OH	C_6=O	C_4-(COOH)$_2$	C_3-(COOH)$_2$	Total[b]
1		1.0	0.08	81.1	9.5	9.4	N. D.[c]	N. D.	> 99
2		2.0	0.19	75.7	12.1	12.2	N. D.	N. D.	> 99
3	130	3.0	0.31	73.4	12.3	14.3	N. D.	N. D.	> 99
4		4.0	0.41	71.9	13.1	15.0	N. D.	N. D.	> 99
5		5.0	0.57	70.9	18.6	10.5	N. D.	N. D.	> 99

6		1.0	0.12	75.4	12.4	12.2	N. D.	N. D.	> 99
7		2.0	0.35	63.6	18.1	18.3	N. D.	N. D.	> 99
8	135	3.0	0.50	71.8	13.1	15.1	N. D.	N. D.	> 99
9		4.0	0.80	68.1	16.0	15.9	N. D.	N. D.	> 99
10		5.0	1.00	36.1	31.0	32.9	N. D.	N. D.	> 99
11		1.0	0.18	65.1	17.5	17.4	N. D.	N. D.	> 99
12		2.0	0.58	60.0	18.9	21.1	N. D.	N. D.	> 99
13	140	3.0	0.97	52.4	21.8	25.8	N. D.	N. D.	> 99
14		4.0	1.39	38.7	29.6	31.7	N. D.	N. D.	> 99
15		5.0	1.74	28.5	33.8	37.7	N. D.	N. D.	> 99

[a] Reaction conditions: Substrate (Cyclohexane, 16.8320 g, 200 mmol), O_2 (1.0 MPa in constant supply), without catalyst, stirring at speed of 600 rpm.

[b] Total selectivity of partial oxidation products (Hydroperoxide, alcohol and ketone).

[c] No detected.

Table S11. Kinetic data in oxidation of cyclohexane catalyzed by Si@Porp. Co.[a]

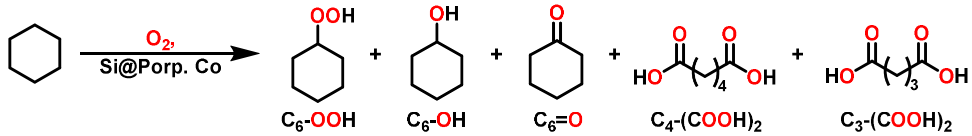

Entry	Temperature (°C)	Reaction time (h)	Conversion (%)	Selectivity (%)					
				C_6-OOH	C_6-OH	C_6=O	C_4-$(COOH)_2$	C_3-$(COOH)_2$	Total[b]
1		4.0	0.46	90.1	N. D.[c]	9.9	N. D.	N. D.	> 99
2		5.0	0.97	90.7	N. D.	9.3	N. D.	N. D.	> 99
3	110	6.0	1.36	80.6	N. D.	17.9	1.4	0.1	98.5
4		7.0	1.75	49.1	19.0	29.9	1.9	0.1	98.0
5		8.0	2.37	49.7	14.9	31.8	2.5	1.1	96.4
6	115	4.0	0.67	89.5	N.D.	10.5	N.D.	N.D.	> 99
7		5.0	1.17	79.3	N. D.	20.7	N. D.	N. D.	> 99

8		6.0	1.76	50.0	6.8	41.8	1.3	0.1	98.6
9		7.0	2.50	36.0	14.0	47.9	2.0	0.1	97.9
10		8.0	3.09	41.5	22.7	32.7	2.8	0.3	96.9
11		4.0	1.56	47.5	11.7	38.3	1.7	0.8	97.5
12		5.0	2.66	64.2	18.7	13.3	2.6	1.2	96.2
13	120	6.0	3.52	15.6	36.7	36.2	7.8	3.7	88.5
14		7.0	4.47	14.8	38.1	37.2	8.3	1.6	90.1
15		8.0	5.05	27.2	32.6	30.0	8.6	1.6	89.8

[a] Reaction conditions: Substrate (Cyclohexane, 16.8320 g, 200 mmol), O_2 (1.0 MPa in constant supply), catalyst (Si@Porp. Co with ratio of metalloporphyrins to Si@NH_2 being 0.16 mmol/g in immobilization, 36 mg, 0.18 mg/mmol), stirring at speed of 600 rpm.

[b] Total selectivity of partial oxidation products (Hydroperoxide, alcohol and ketone).

[c] No detected.

Table S12. Kinetic data in oxidation of cyclohexane catalyzed by Si@Porp. Co&Cu.[a]

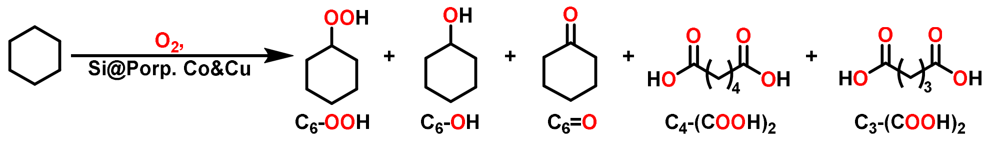

Entry	Temperature (°C)	Reaction time (h)	Conversion (%)	Selectivity (%)					
				C_6-OOH	C_6-OH	C_6=O	C_4-$(COOH)_2$	C_3-$(COOH)_2$	Total[b]
1		4.0	0.47	84.0	N. D.[c]	16.0	N. D.	N. D.	> 99
2		5.0	1.08	72.0	N. D.	25.6	2.2	0.2	97.6
3	110	6.0	1.57	51.3	7.1	39.3	2.2	0.1	97.7
4		7.0	1.89	47.7	1.9	48.1	2.2	0.1	97.7
5		8.0	2.52	47.8	1.9	42.4	6.7	1.2	92.1
6	115	4.0	0.67	81.6	N. D.	18.4	N. D.	N. D.	> 99
7		5.0	1.28	66.8	N.D.	30.8	2.3	0.1	97.6

8		6.0	1.97	64.3	6.8	26.7	2.1	0.1	97.8
9		7.0	2.65	45.2	6.6	45.8	2.4	N. D.	97.6
10		8.0	3.36	42.2	14.6	40.6	2.5	0.1	97.4
11		4.0	1.77	50.7	24.1	21.4	2.4	1.4	96.2
12		5.0	2.66	50.4	26.6	21.1	1.4	0.5	98.1
13	120	6.0	3.72	20.3	12.7	62.5	3.5	1.0	95.5
14		7.0	4.50	13.0	44.5	38.9	3.0	0.6	96.4
15		8.0	5.27	15.8	41.9	34.6	6.2	1.5	92.3

[a] Reaction conditions: Substrate (Cyclohexane, 16.8320 g, 200 mmol), O_2 (1.0 MPa in constant supply), catalyst (Si@Porp. Co&Cu with molar ratio of Co and Cu being 1:2, and in immobilization, ratio of metalloporphyrins to Si@NH_2 being 0.16 mmol/g, 32 mg, 0.16 mg/mmol), stirring at speed of 600 rpm.

[b] Total selectivity of partial oxidation products (Hydroperoxide, alcohol and ketone).

[c] No detected.

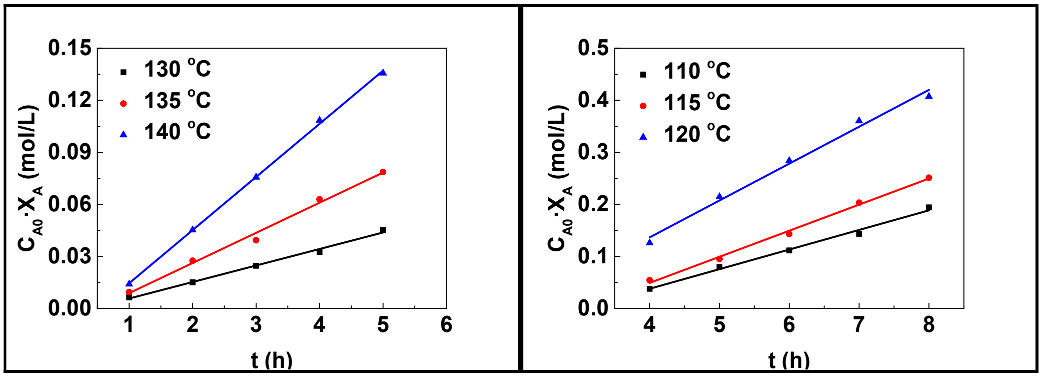

a b

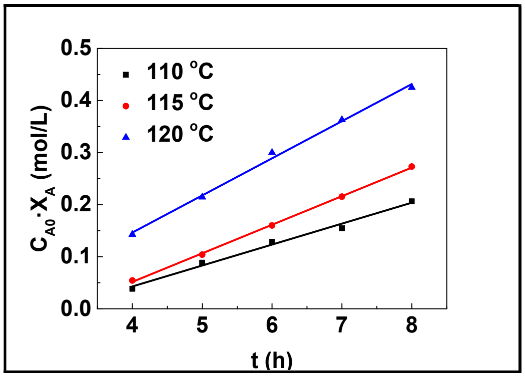

c

Figure S3. Pseudo-zero-order fits for oxidation of cyclohexane with O_2 without catalyst (a), and catalyzed by Si@Porp. Co (b) and Si@Porp. Co&Cu (c). Reaction conditions: Substrate (Cyclohexane, 16.8320 g, 200 mmol), O_2 (1.0 MPa in constant supply), catalyst (Si@Porp. Co, 36 mg, Si@Porp. Co&Cu, 32 mg), stirring at speed of 600 rpm.

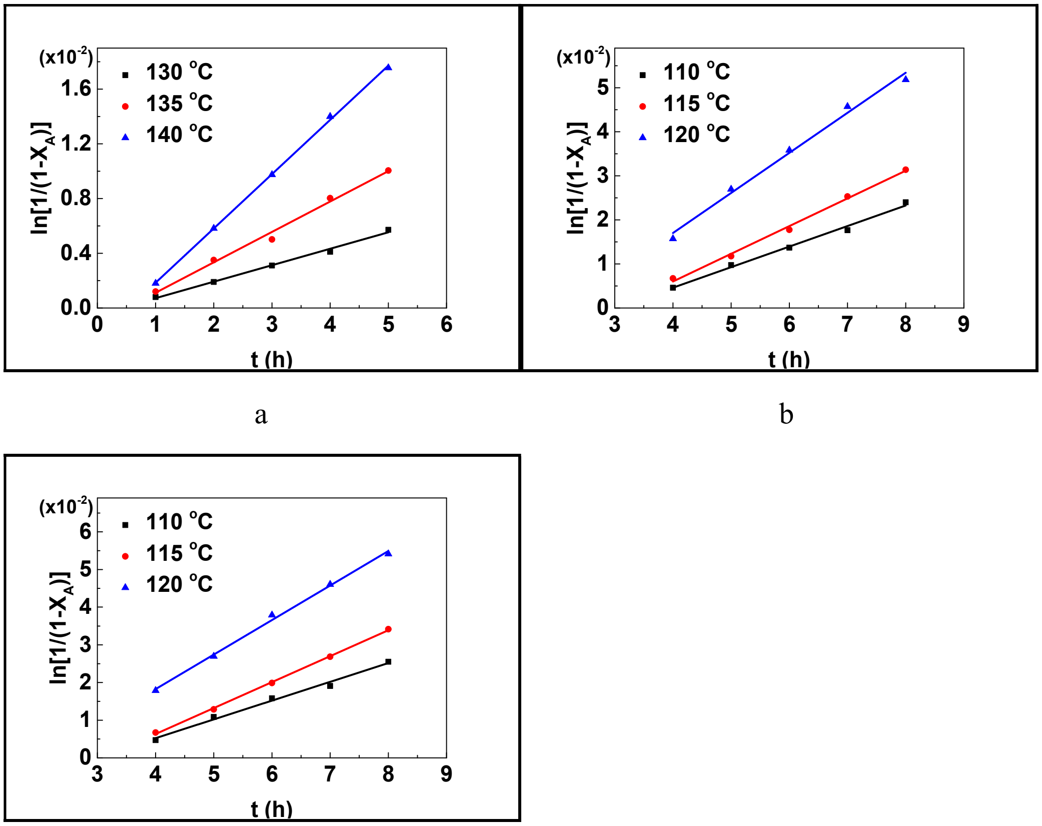

c

Figure S4. Pseudo-first-order fits for oxidation of cyclohexane with O_2 without catalyst (a), and catalyzed by Si@Porp. Co (b) and Si@Porp. Co&Cu (c). Reaction conditions: Substrate (Cyclohexane, 16.8320 g, 200 mmol), O_2 (1.0 MPa in constant supply), catalyst (Si@Porp. Co, 36 mg, Si@Porp. Co&Cu, 32 mg), stirring at speed of 600 rpm.

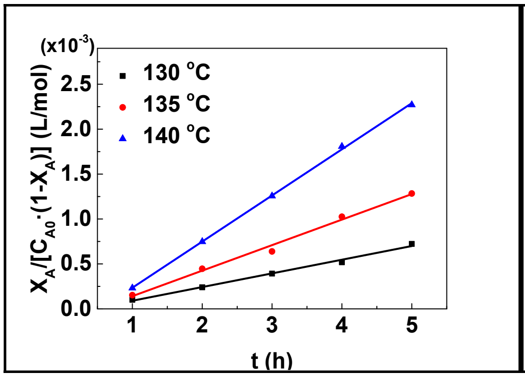

a

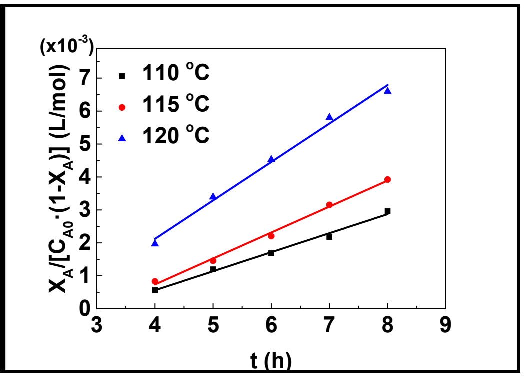

b

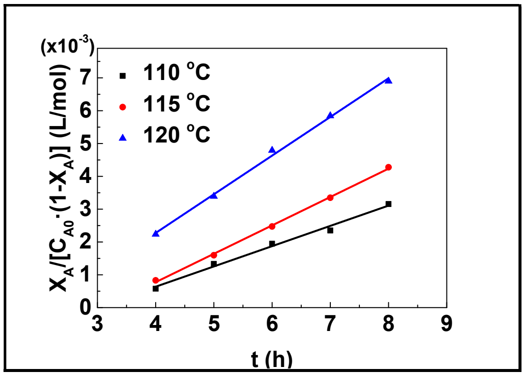

c

Figure S5. Pseudo-second-order fits for oxidation of cyclohexane with O_2 without catalyst (a), and catalyzed by Si@Porp. Co (b) and Si@Porp. Co&Cu (c). Reaction conditions: Substrate (Cyclohexane, 16.8320 g, 200 mmol), O_2 (1.0 MPa in constant supply), catalyst (Si@Porp. Co, 36 mg, Si@Porp. Co&Cu, 32 mg), stirring at speed of 600 rpm.

Table S13. The pseudo-zero-order kinetic parameters in oxidation of cyclohexane[a].

Entry	Catalysts	Temperature (°C)	k ($mol\cdot L^{-1}\cdot h^{-1}$)	R^2
1	/	130	0.0095	0.9919
2		135	0.0174	0.9894

3		140	0.0307	0.9992
4		110	0.0377	0.9901
5	Si@Porp. Co	115	0.0502	0.9946
6		120	0.0709	0.9872
7		110	0.0402	0.9870
8	Si@Porp. Co&Cu	115	0.0549	0.9991
9		120	0.0713	0.9949

[a] Reaction conditions: Substrate (Cyclohexane, 16.8320 g, 200 mmol), O_2 (1.0 MPa in constant supply), catalyst (Si@Porp. Co, 36 mg, Si@Porp. Co&Cu, 32 mg), stirring at speed of 600 rpm.

Table S14. The pseudo-first-order kinetic parameters in oxidation of cyclohexane[a].

Entry	Catalysts	Temperature (ºC)	k (h^{-1})	R^2
1		130	0.1203	0.9918
2	/	135	0.2222	0.9892
3		140	0.3968	0.9992
4		110	0.4666	09897
5	Si@Porp. Co	115	0.6288	0.9942
6		120	0.9096	0.9883
7		110	0.4985	0.9872
8	Si@Porp. Co&Cu	115	0.6889	0.9988
9		120	0.9165	0.9956

[a] Reaction conditions: Substrate (Cyclohexane, 16.8320 g, 200 mmol), O_2 (1.0 MPa in

constant supply), catalyst (Si@Porp. Co, 36 mg, Si@Porp. Co&Cu, 32 mg), stirring at speed of 600 rpm.

Table S15. The pseudo-second-order kinetic parameters in oxidation of cyclohexane[a].

Entry	Catalysts	Temperature (ºC)	k ($L \cdot mol^{-1} \cdot h^{-1}$)	R^2
1		130	0.1522	0.9917
2	/	135	0.2841	0.9892
3		140	0.5137	0.9993
4		110	0.5776	0.9893
5	Si@Porp. Co	115	0.7883	0.9938
6		120	1.1673	0.9894
7		110	0.6176	0.9872
8	Si@Porp. Co&Cu	115	0.8647	0.9985
9		120	1.1781	0.9961

[a] Reaction conditions: Substrate (Cyclohexane, 16.8320 g, 200 mmol), O_2 (1.0 MPa in constant supply), catalyst (Si@Porp. Co, 36 mg, Si@Porp. Co&Cu, 32 mg), stirring at speed of 600 rpm.

5. Mechanism study

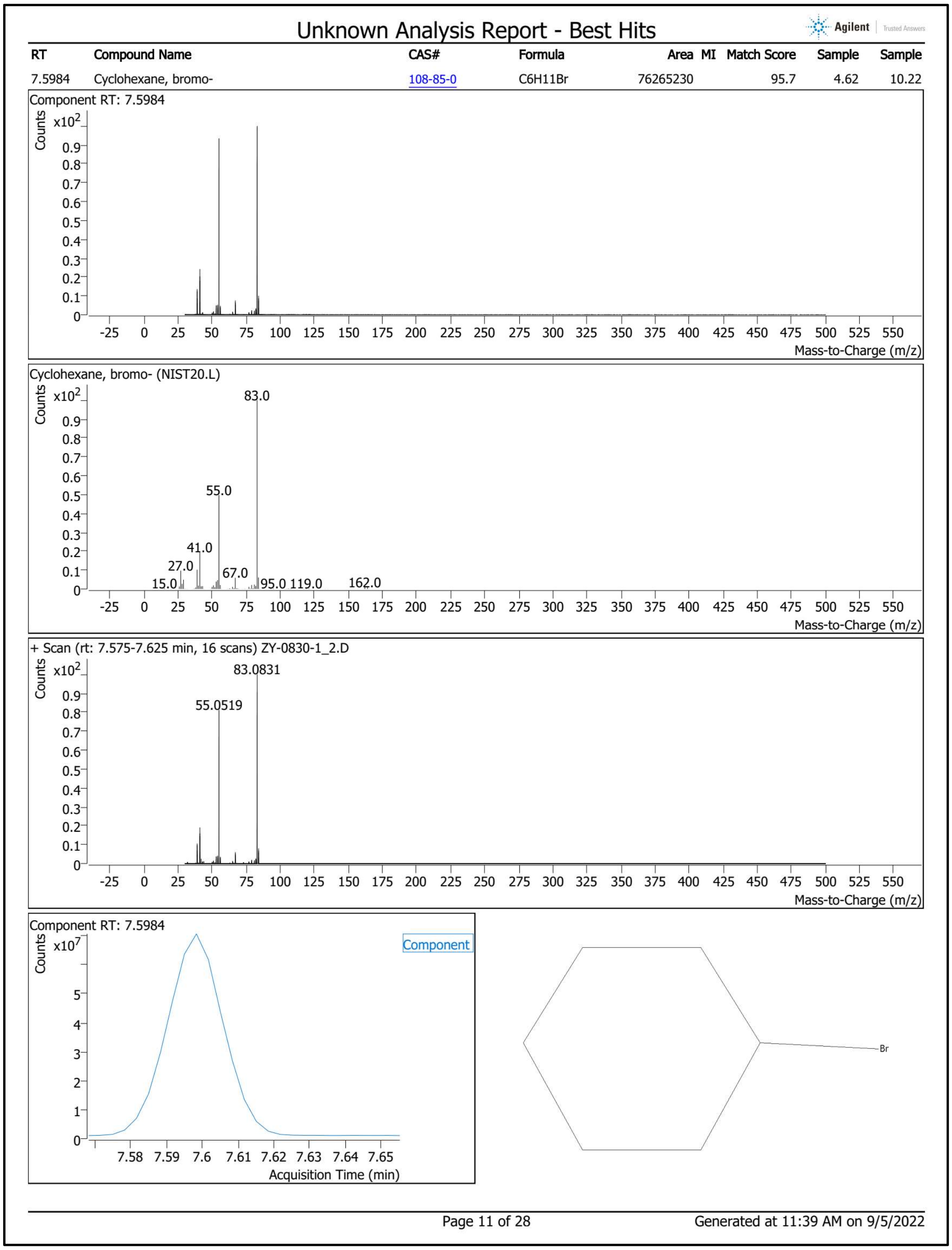

Figure S6. GC-MS spectra of oxidation of cyclohexane with O_2 catalyzed by Si@Porp. Co&Cu in the presence of $CBrCl_3$ as quenching reagent.

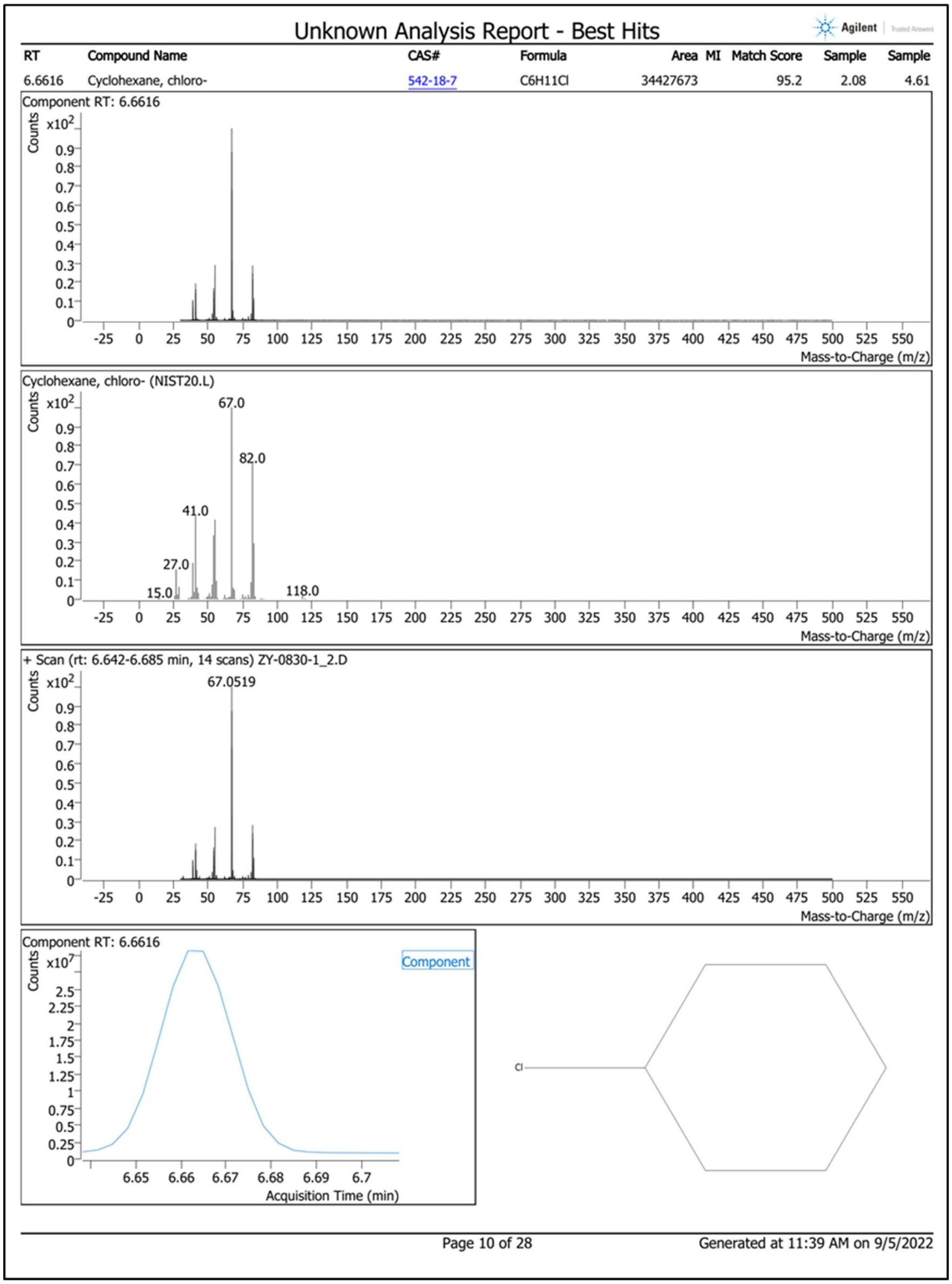

Figure S7. GC-MS spectra of oxidation of cyclohexane with O_2 catalyzed by Si@Porp. Co&Cu in the presence of $CBrCl_3$ as quenching reagent.

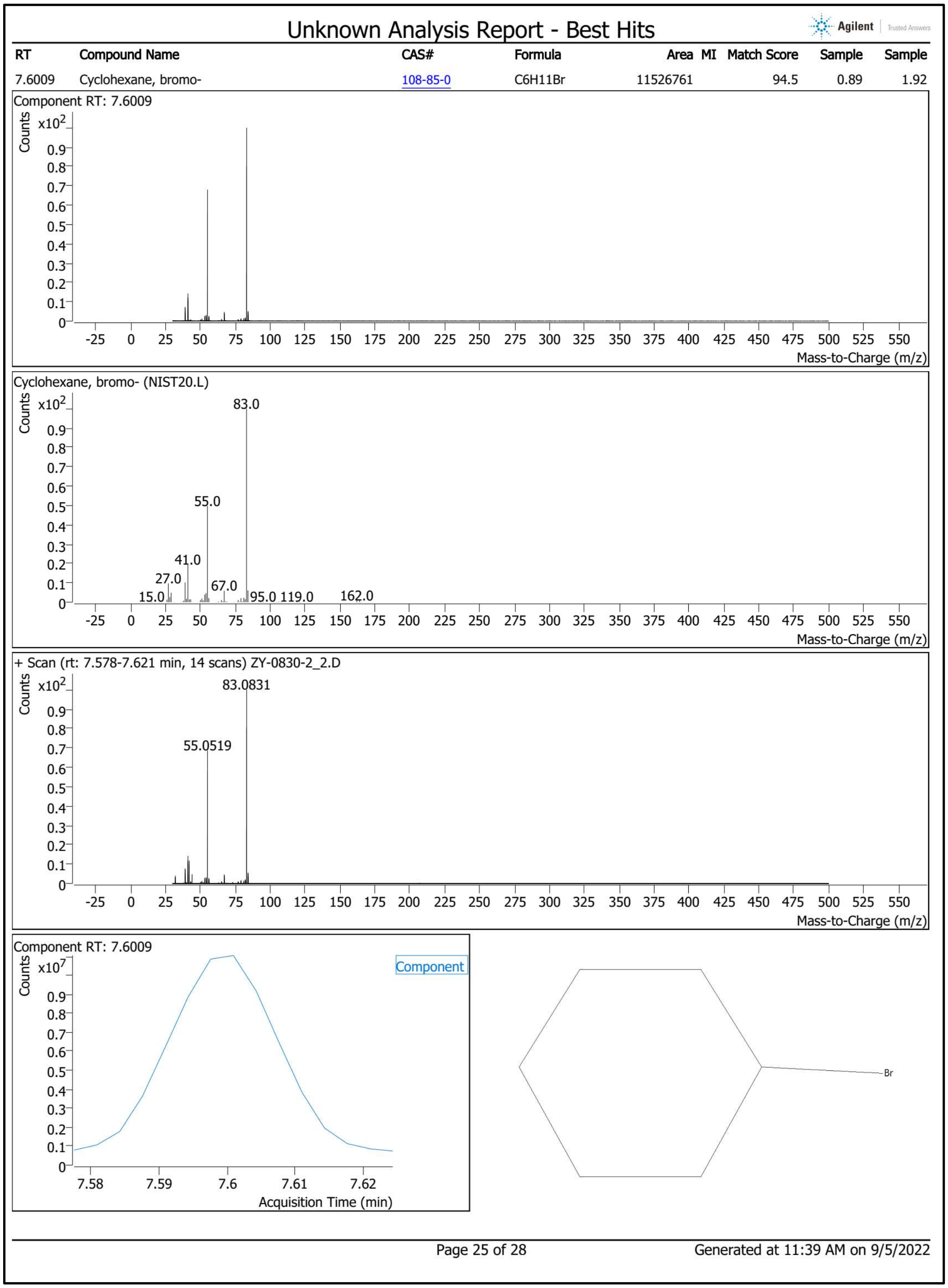

Figure S8. GC-MS spectra of oxidation of cyclohexane with O_2 catalyzed by Si@Porp. Co&Cu in the presence of *t*-BuBr as quenching reagent.

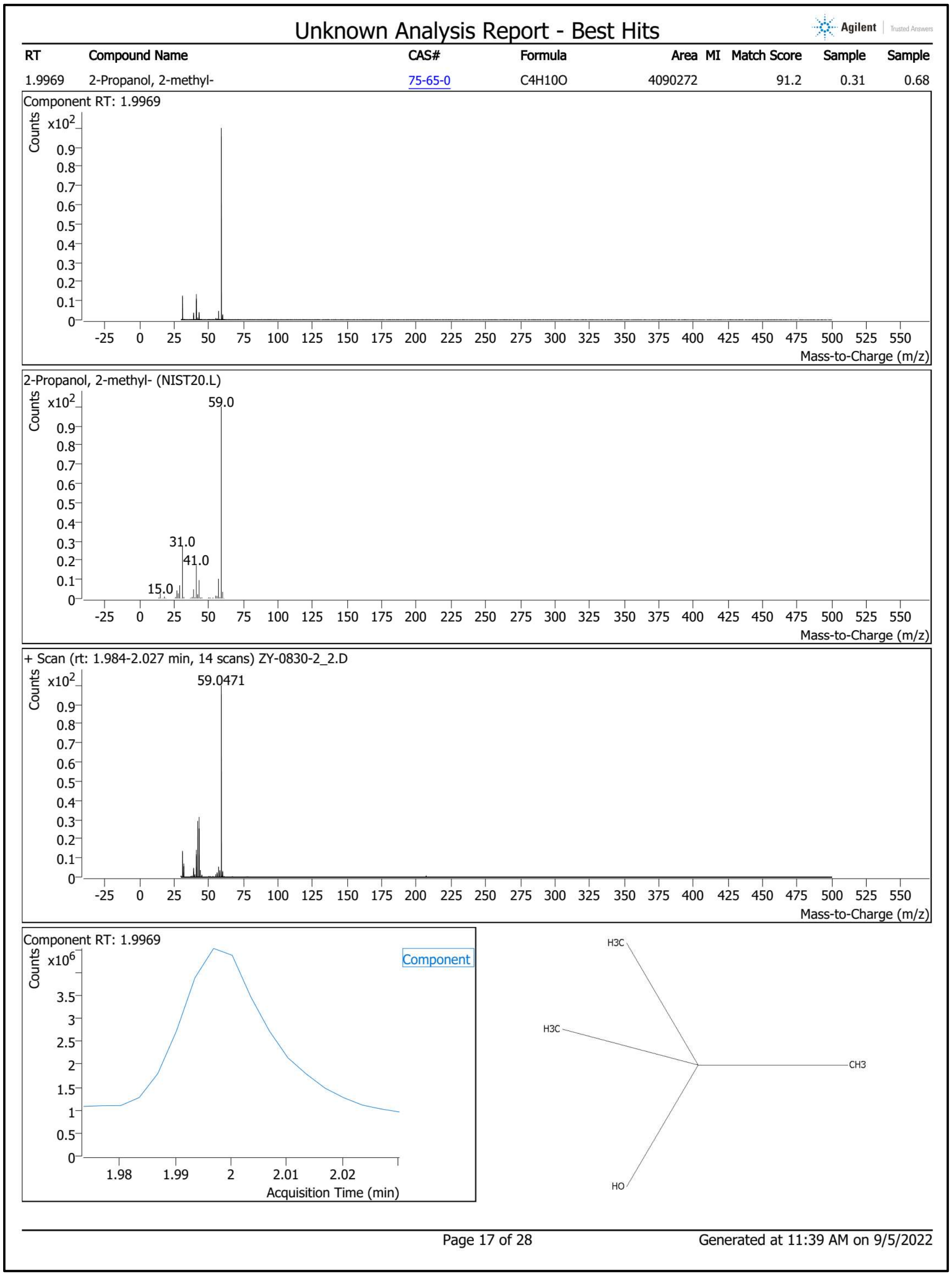

Figure S9. GC-MS spectra of oxidation of cyclohexane with O_2 catalyzed by Si@Porp. Co&Cu in the presence of *t*-BuBr as quenching reagent.

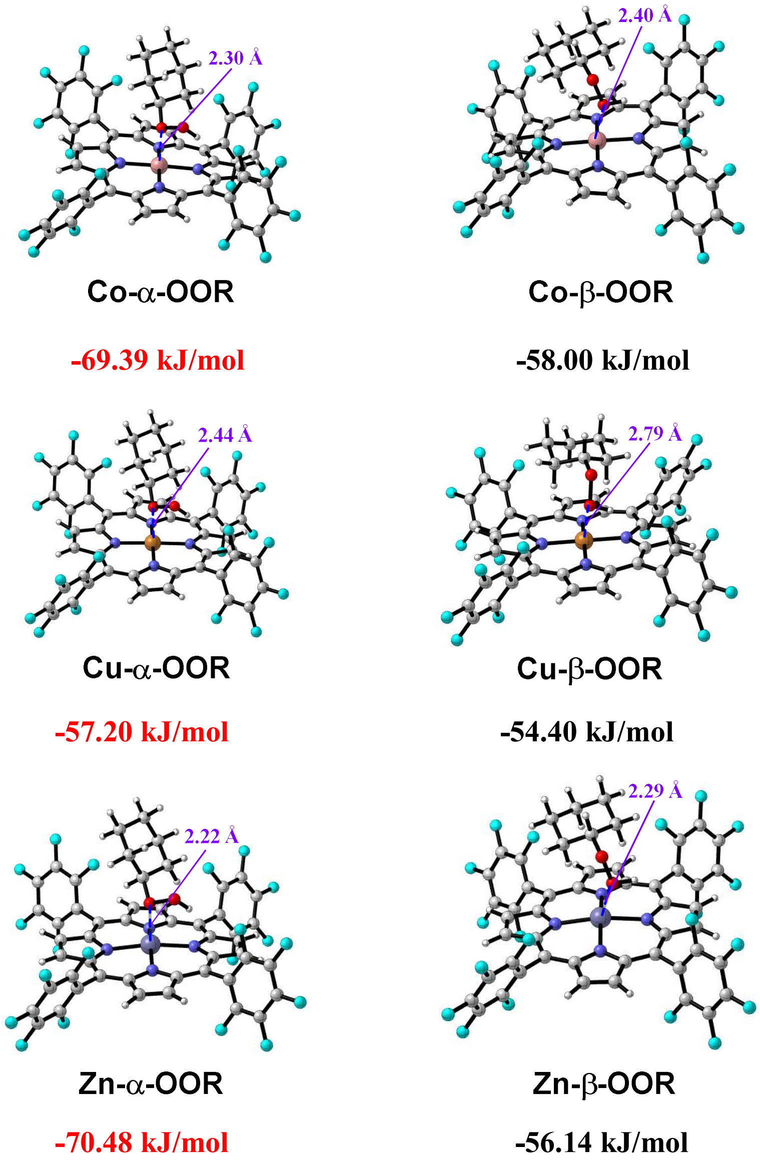

Figure S10. Binding mode between T(perF)PPCo and C_6H_{11}-OOH, T(perF)PPCu and C_6H_{11}-OOH, T(perF)PPZn and C_6H_{11}-OOH, and corresponding binding energies obtained from quantum chemical calculation.

6. ^{1}H NMR spectra of porphyrins

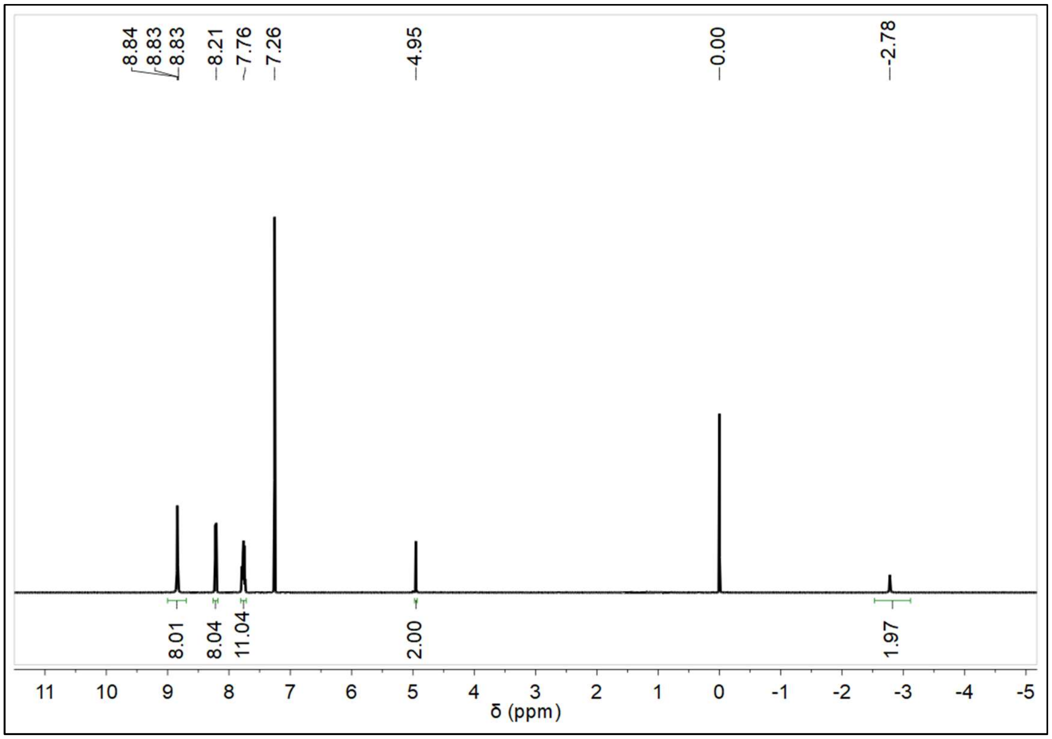

Figure S11. ^{1}H NMR spectrum of TrisP(4-CH_2Cl)PP.

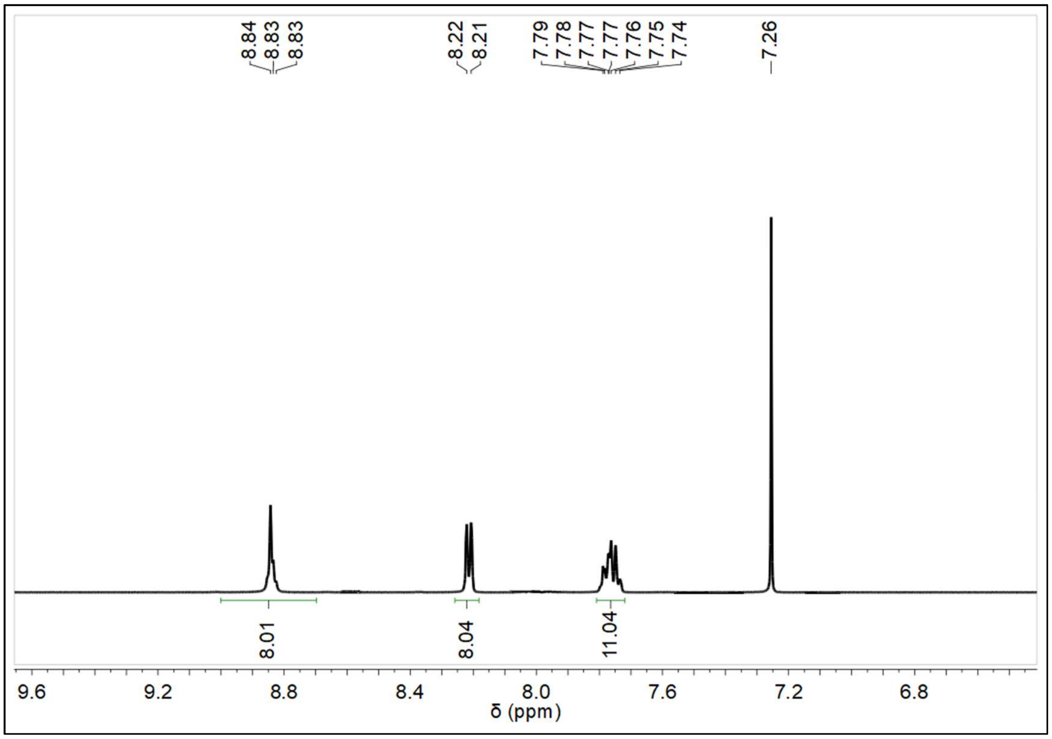

Figure S12. Locally magnified ^{1}H NMR spectrum of TrisP(4-CH_2Cl)PP.

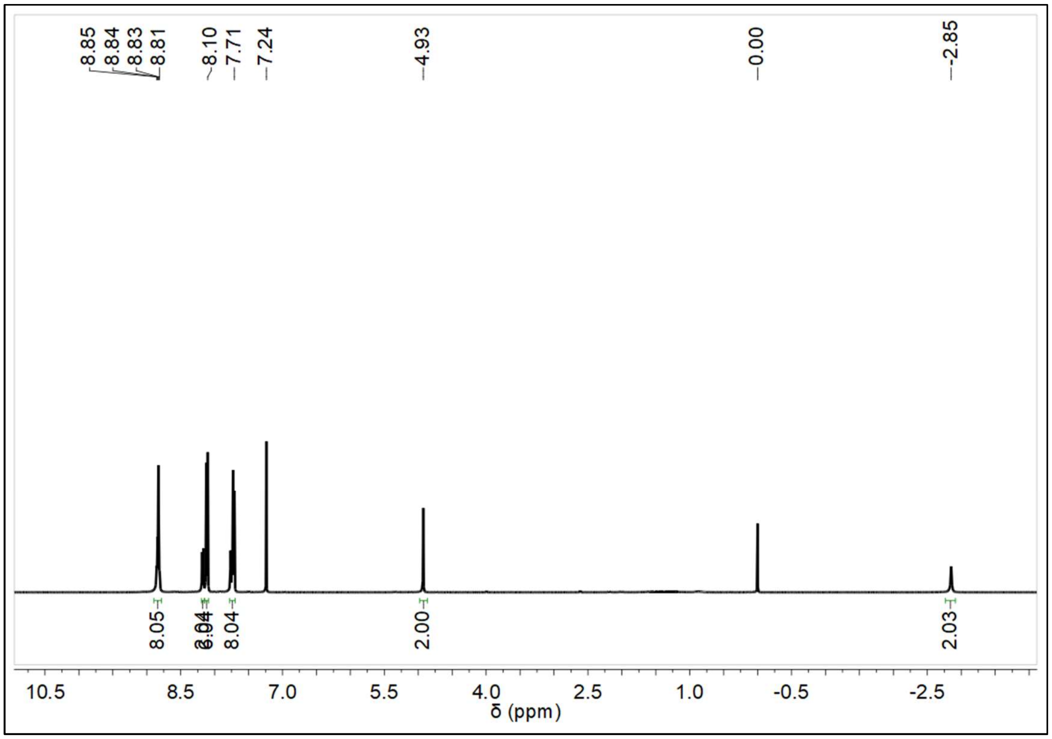

Figure S13. ^{1}H NMR spectrum of Tris(4-Cl)P(4-CH_2Cl)PP.

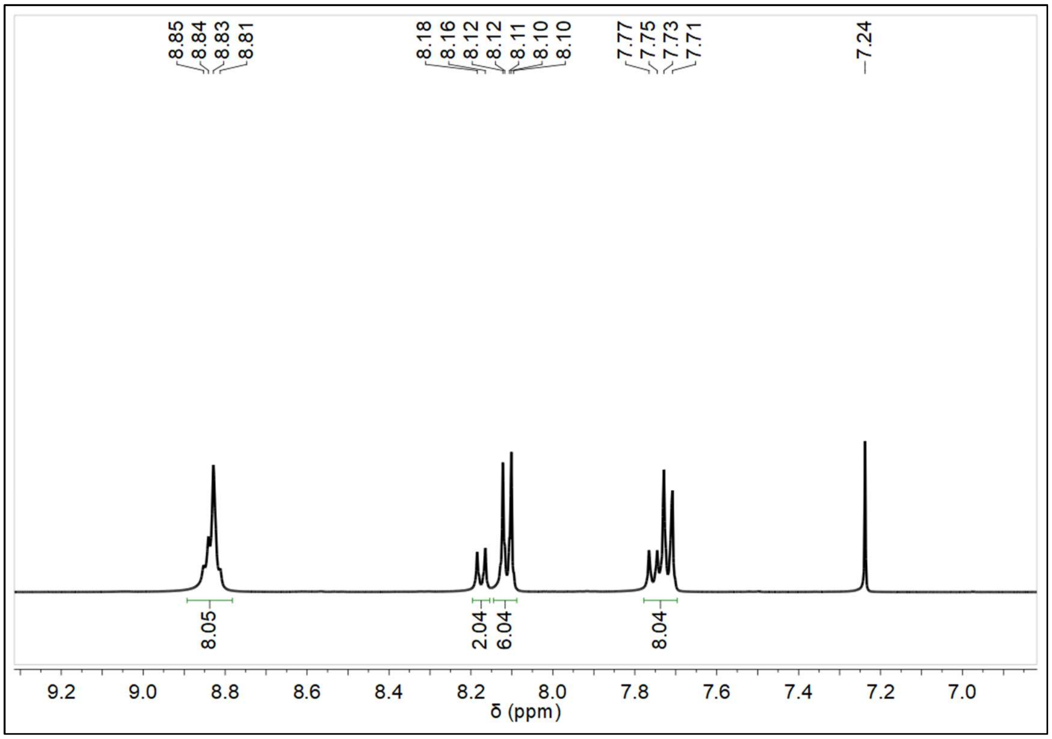

Figure S14. Locally magnified ^{1}H NMR spectrum of Tris(4-ClP(4-CH_2Cl)PP.

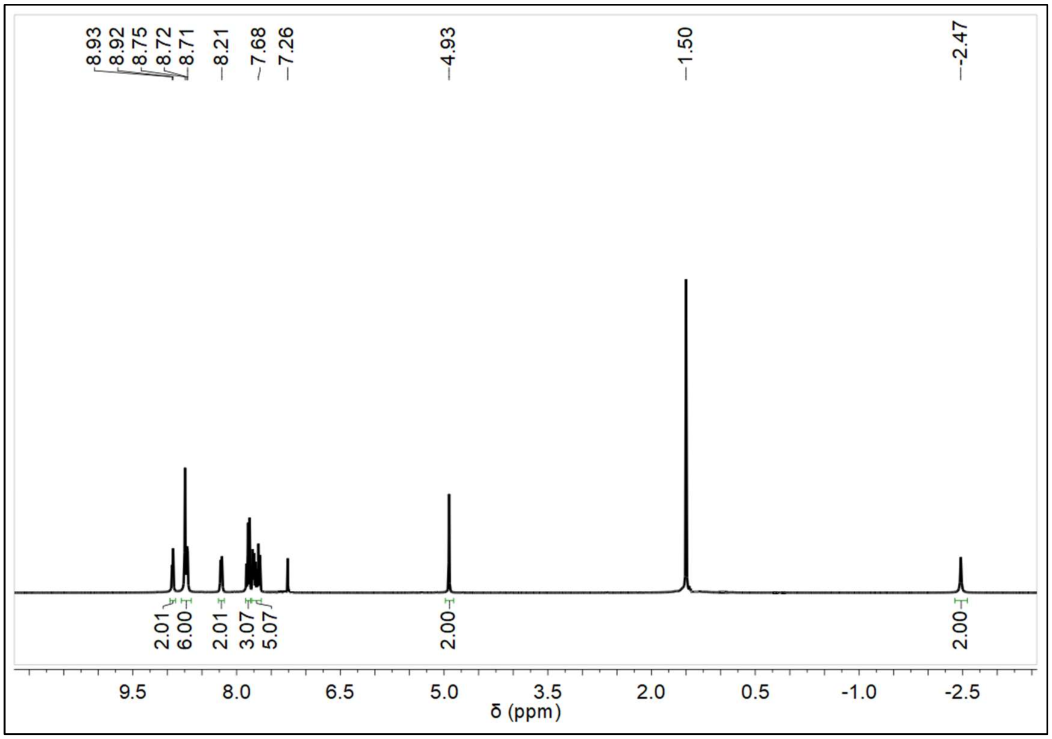

Figure S15. ^{1}H NMR spectrum of Tris(2,3,6-triCl)P(4-CH_2Cl)PP.

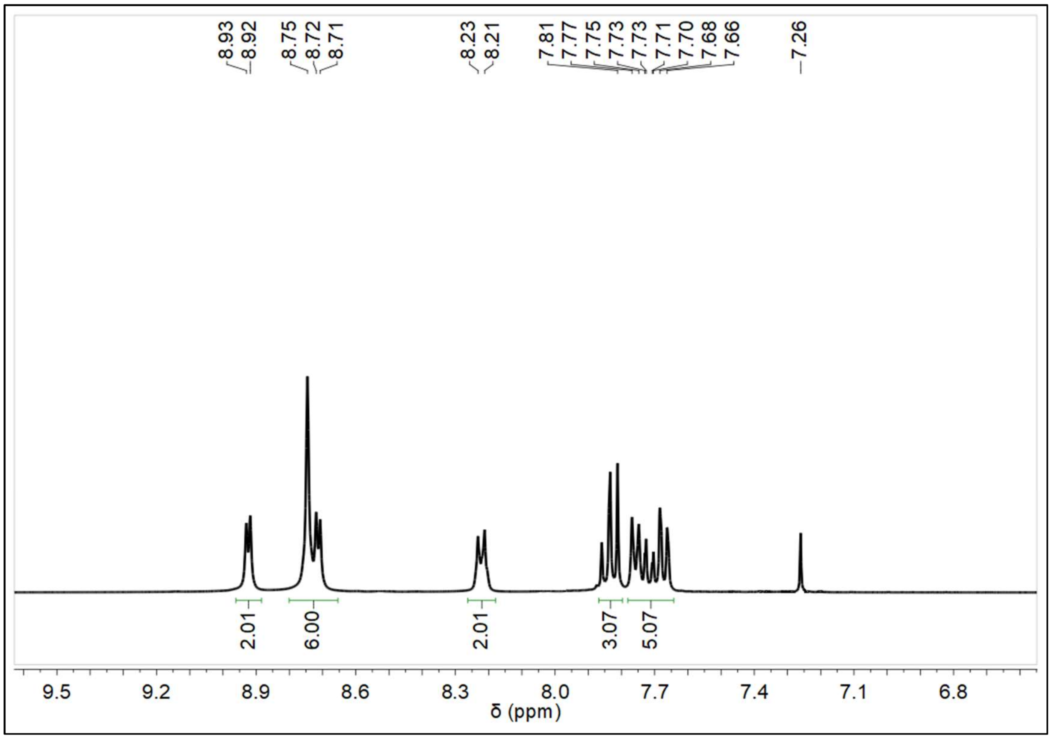

Figure S16. Locally magnified ^{1}H NMR spectrum of Tris(2,3,6-triCl)P(4-CH_2Cl)PP.

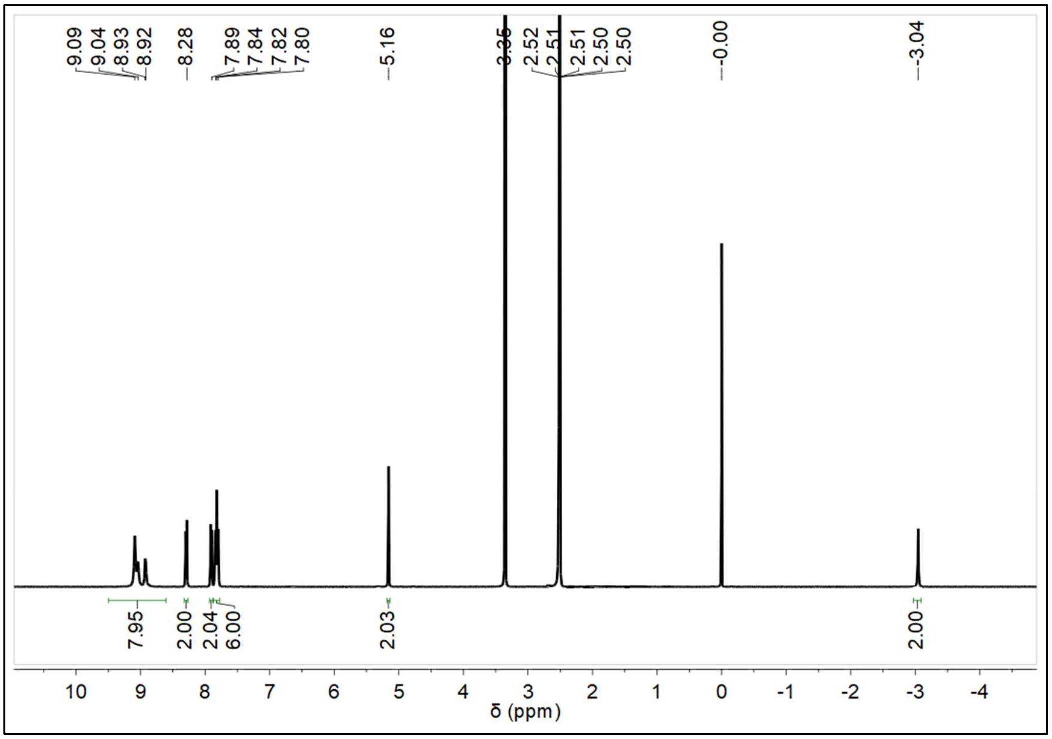

Figure S17. ^{1}H NMR spectrum of Tris(2,4,6-triF)P(4-CH_2Cl)PP.

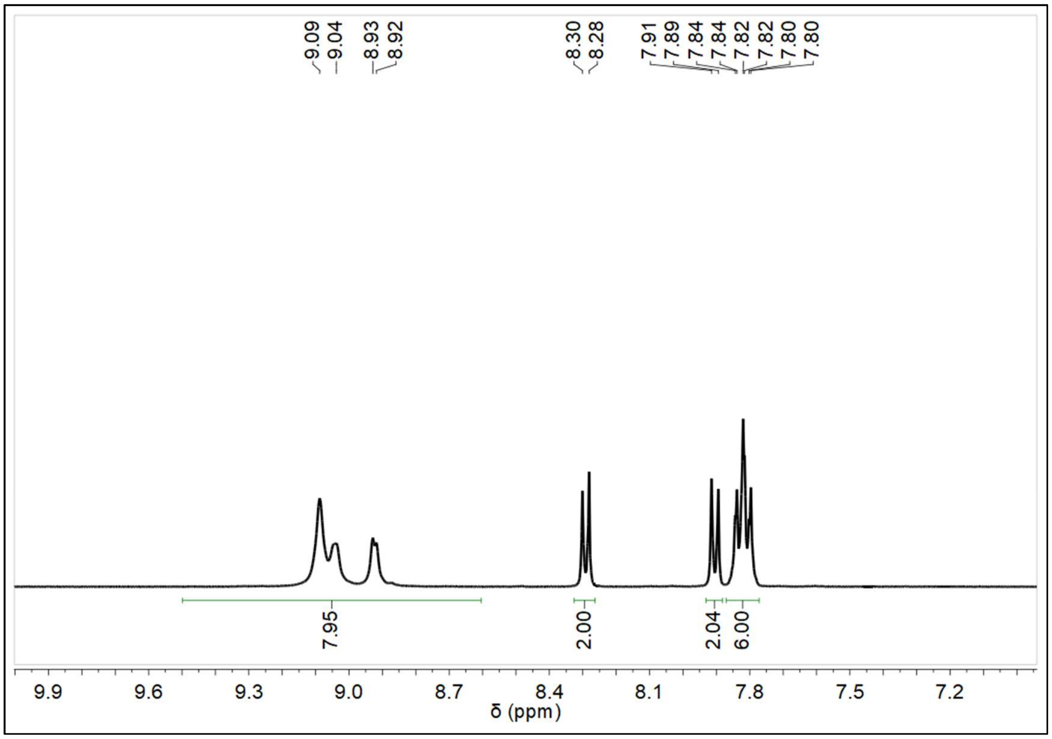

Figure S18. Locally magnified ^{1}H NMR spectrum of Tris(2,4,6-triF)P(4-CH_2Cl)PP.

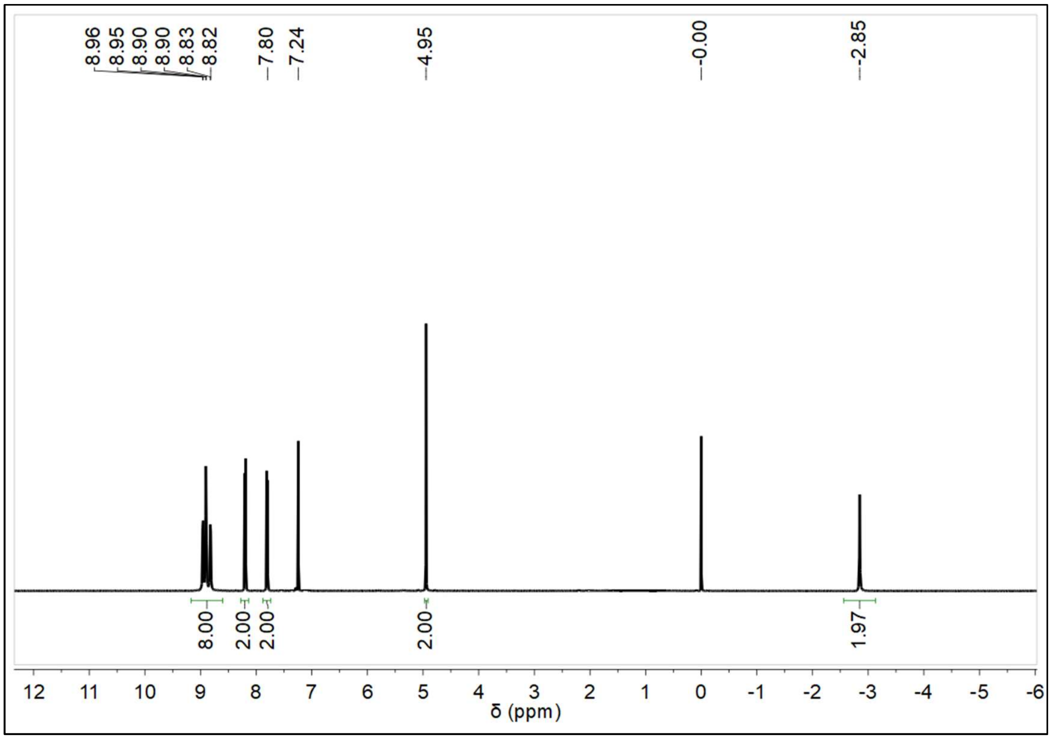

Figure S19. ^{1}H NMR spectrum of Tris(perF)P(4-CH_2Cl)PP.

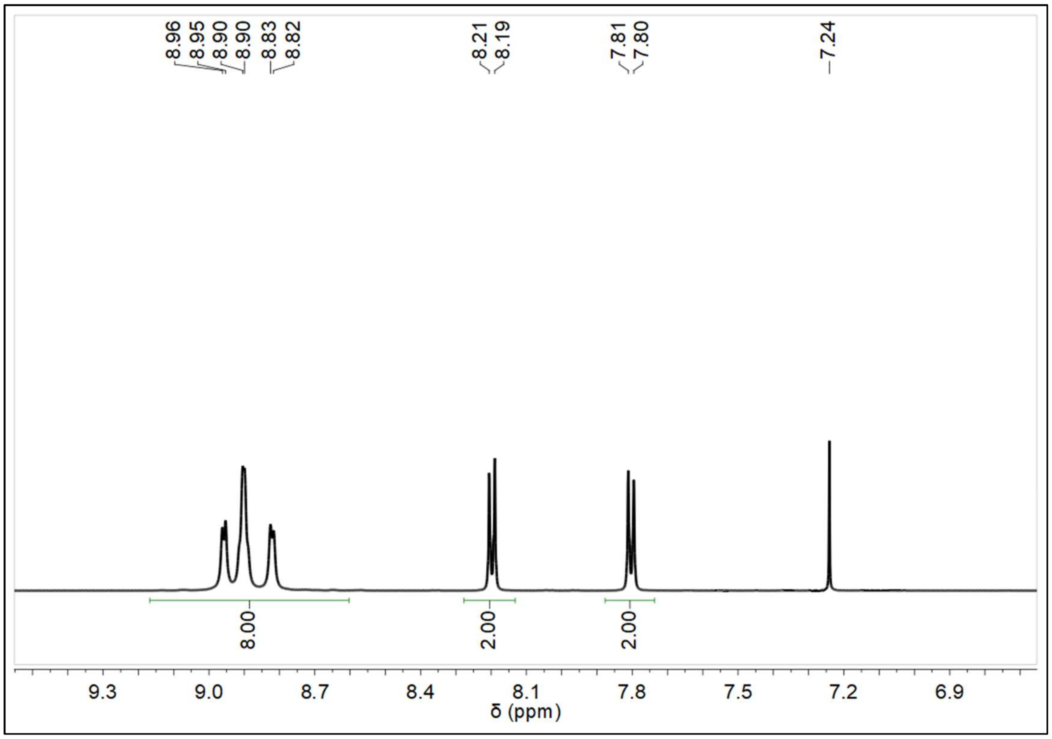

Figure S20. Locally magnified ^{1}H NMR spectrum of Tris(perF)P(4-CH_2Cl)PP.

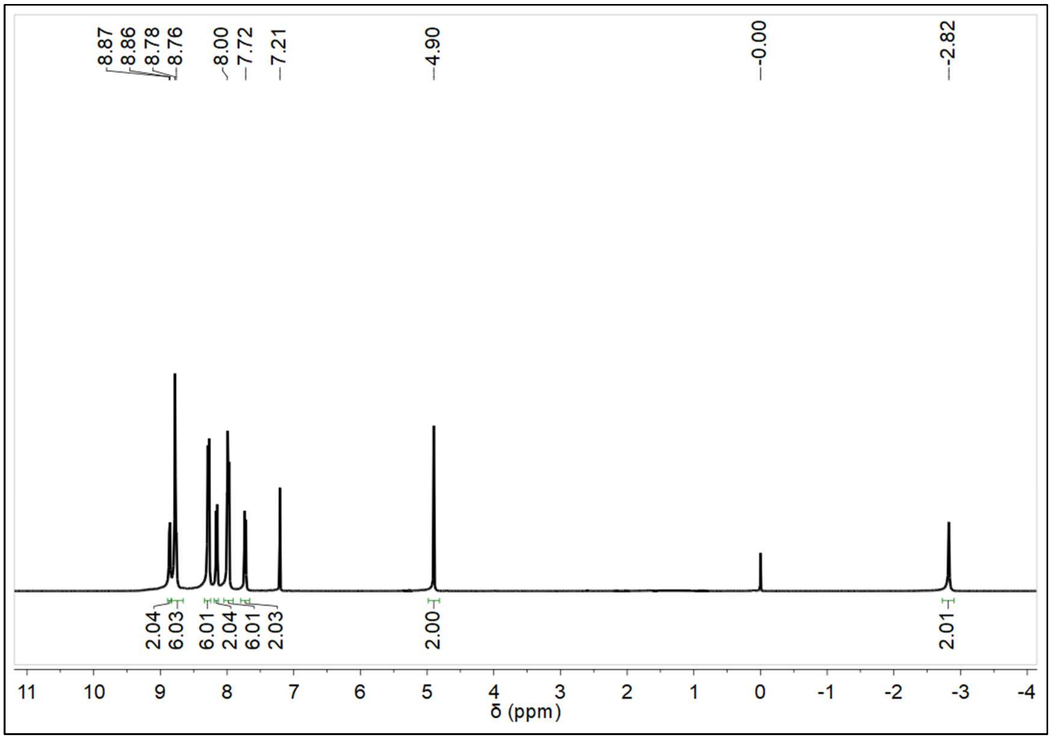

Figure S21. ^{1}H NMR spectrum of Tris(4-CF_3)P(4-CH_2Cl)PP.

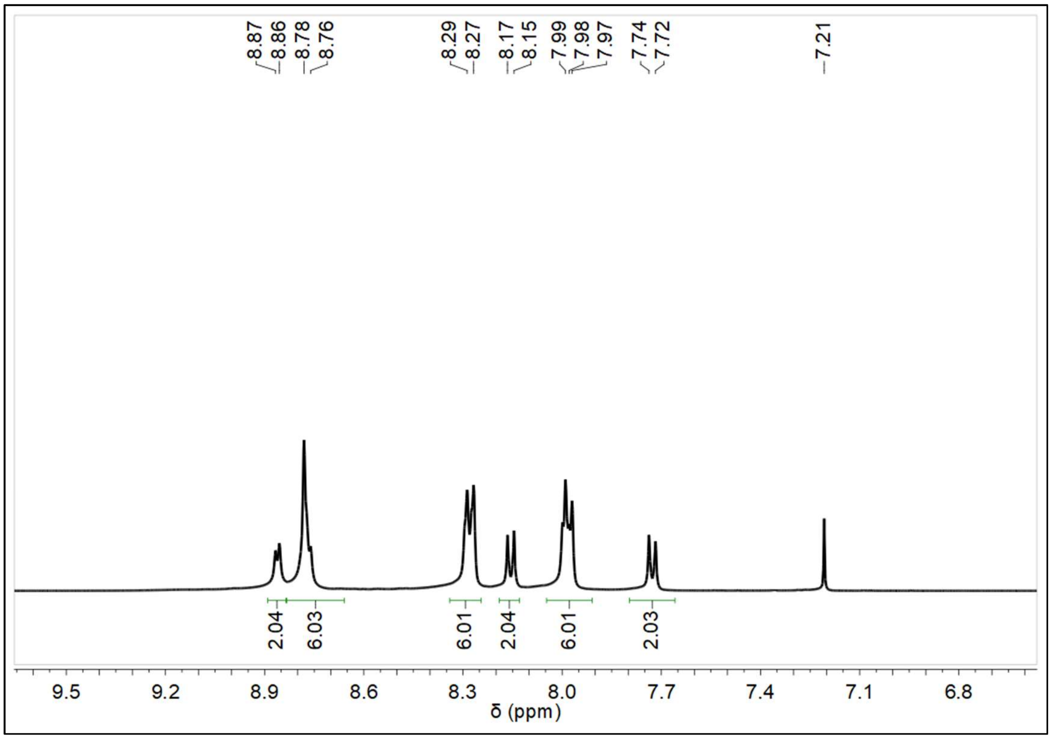

Figure S22. Locally magnified ^{1}H NMR spectrum of Tris(4-CF_3)P(4-CH_2Cl)PP.

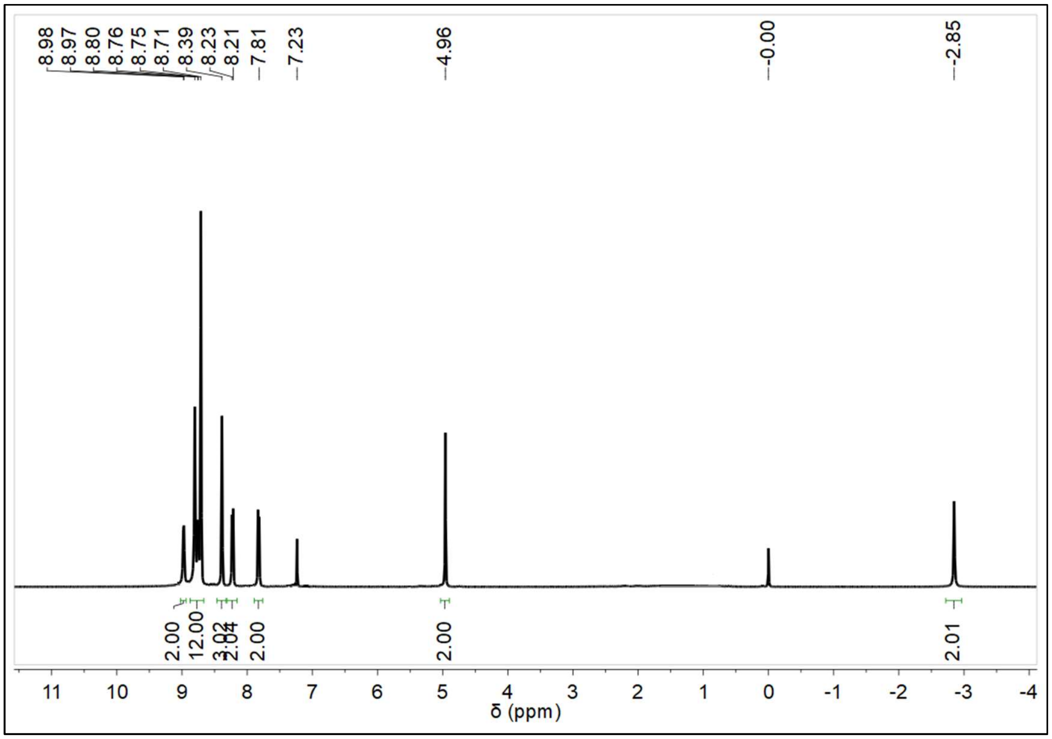

Figure S23. ^{1}H NMR spectrum of Tris(3,5-diCF_3)P(4-CH_2Cl)PP.

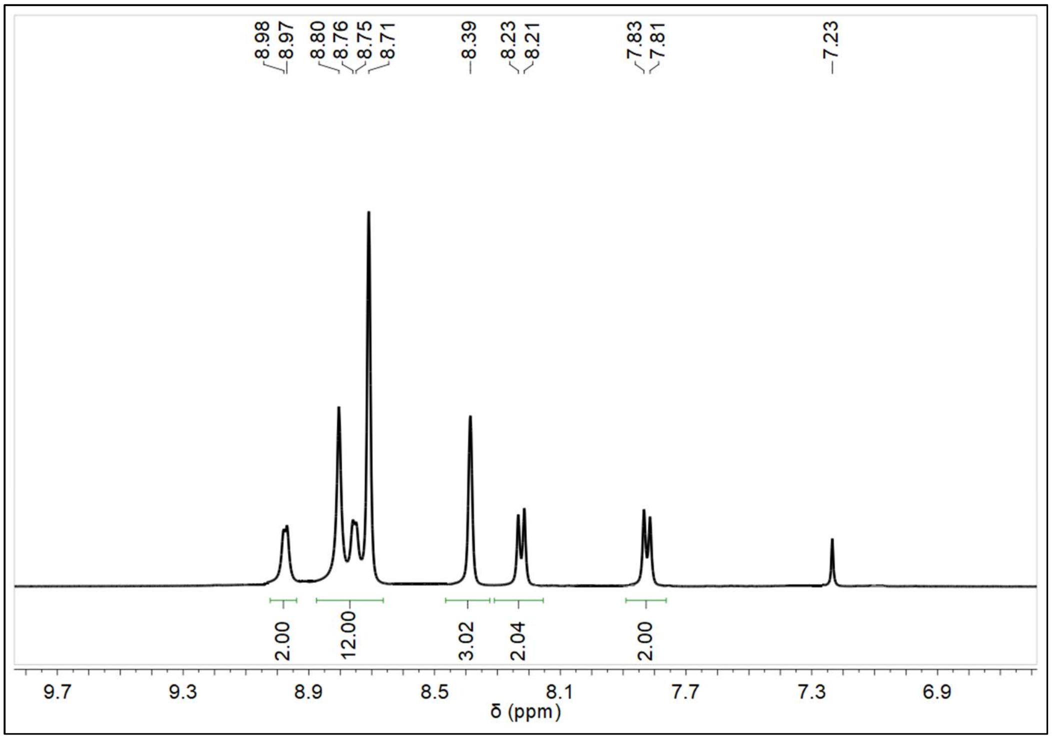

Figure S24. Locally magnified ^{1}H NMR spectrum of Tris(3,5-diCF_3)P(4-CH_2Cl)PP.

7. ^{13}C NMR spectra of porphyrins

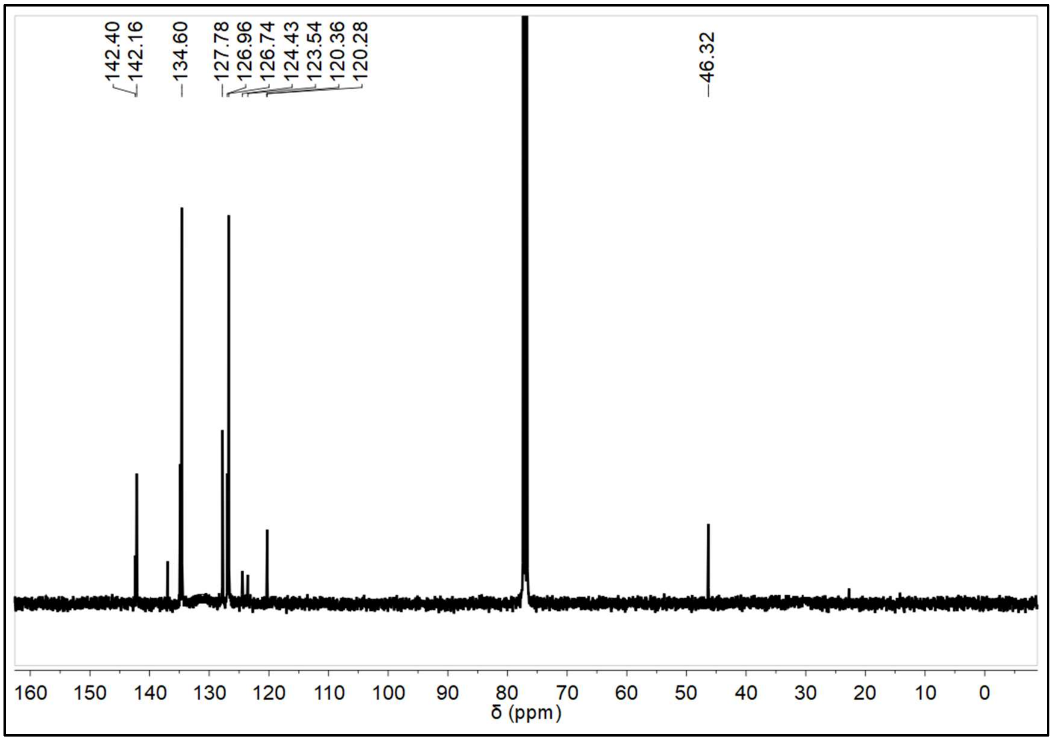

Figure S25. ^{13}C NMR spectrum of TrisP(4-CH_2Cl)PP.

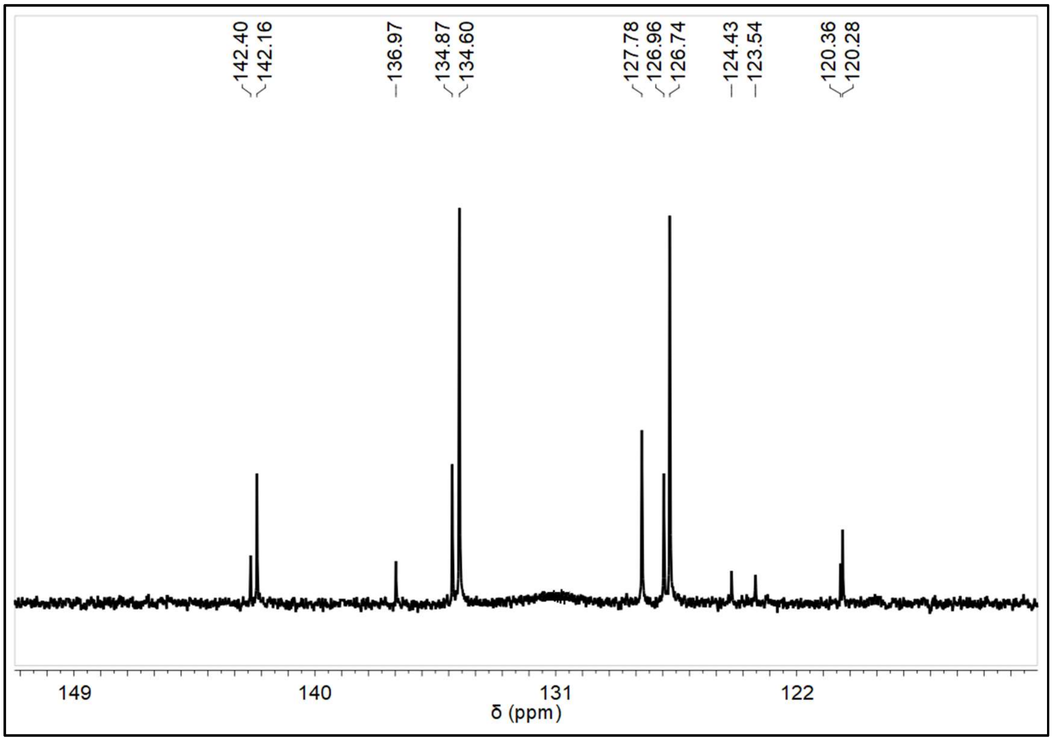

Figure S26. Locally magnified ^{13}C NMR spectrum of TrisP(4-CH_2Cl)PP.

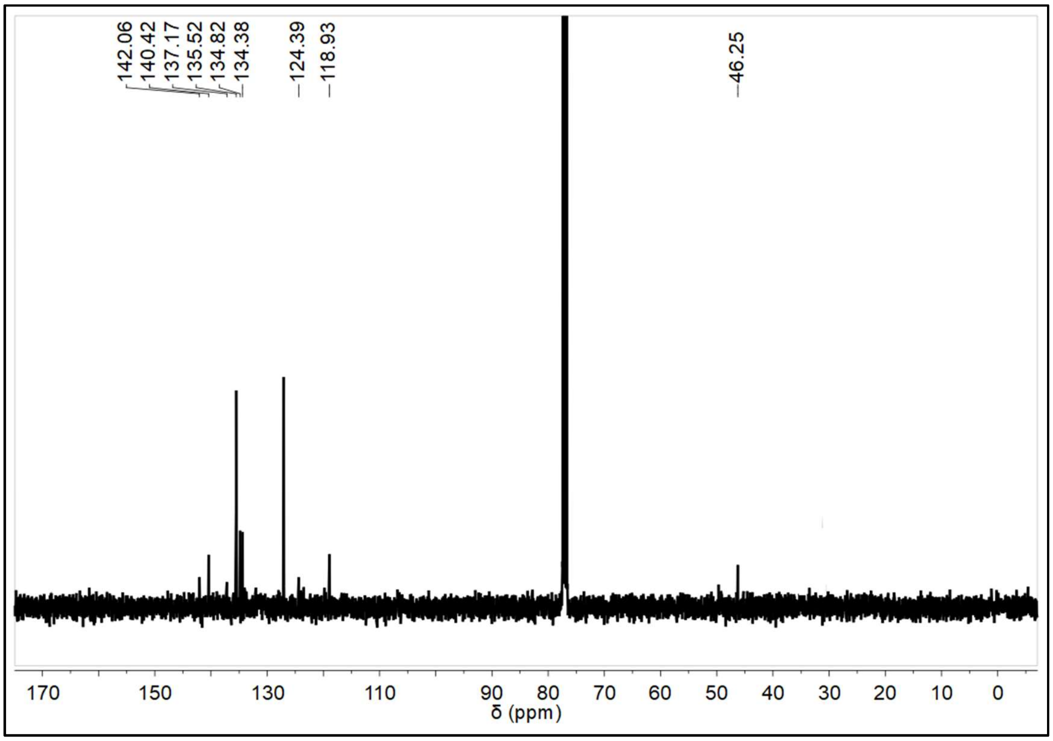

Figure S27. ^{13}C NMR spectrum of Tris(4-Cl)P(4-CH_2Cl)PP.

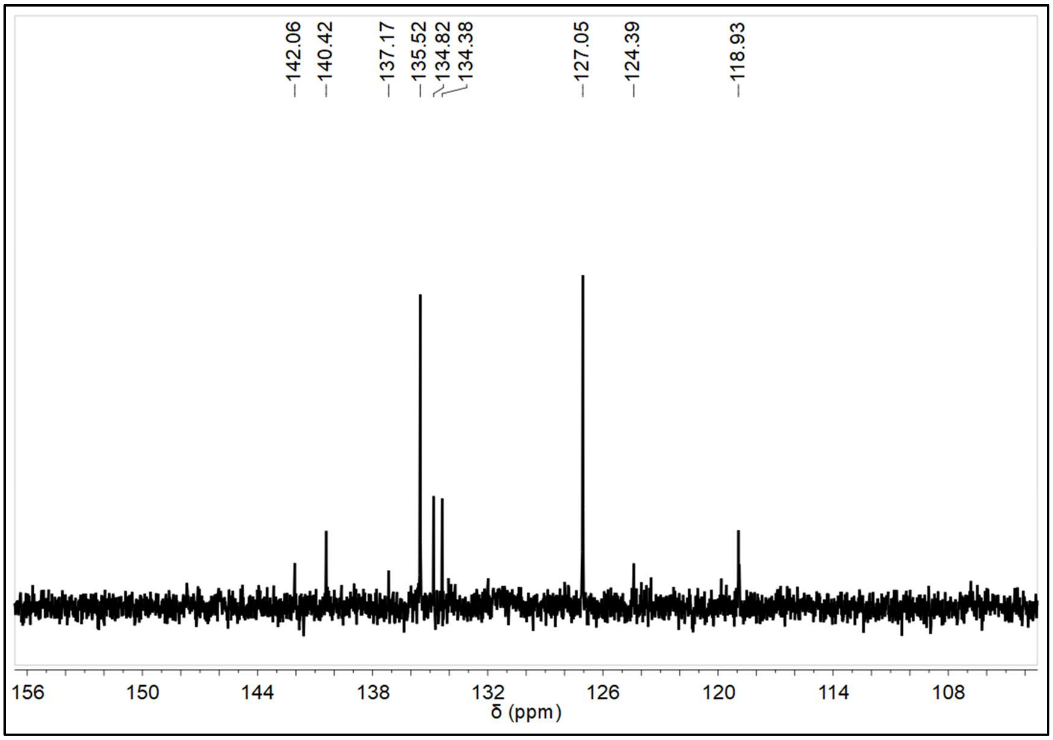

Figure S28. Locally magnified ^{13}C NMR spectrum of Tris(4-Cl)P(4-CH_2Cl)PP.

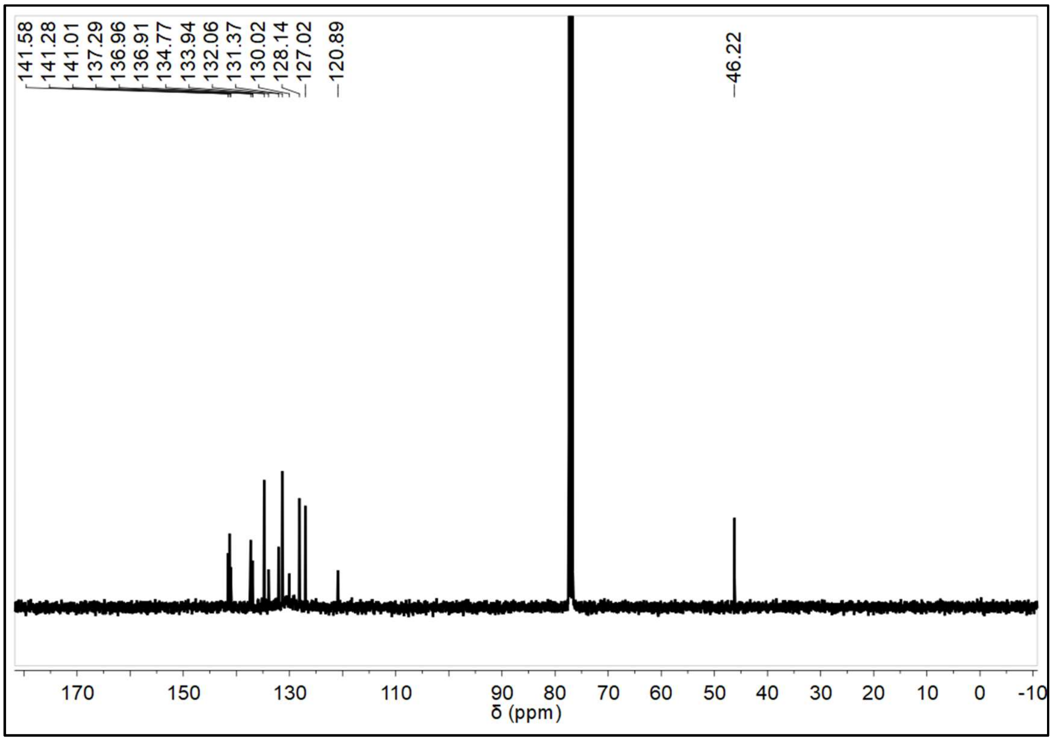

Figure S29. ^{13}C NMR spectrum of Tris(2,3,6-triCl)P(4-CH_2Cl)PP.

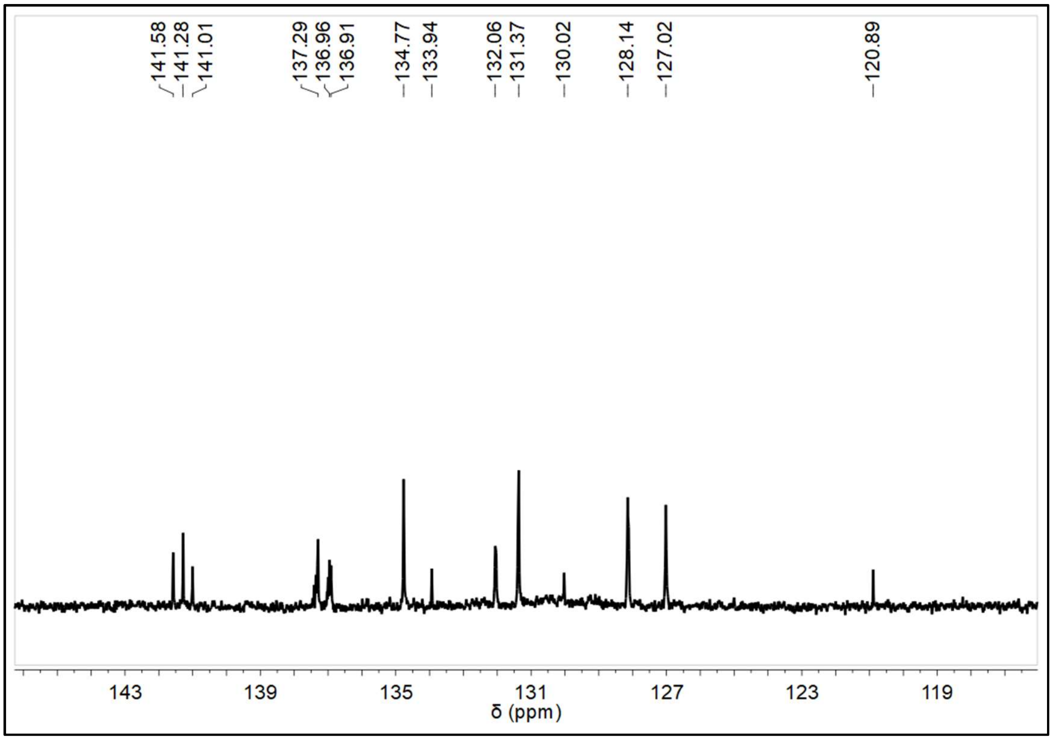

Figure S30. Locally magnified ^{13}C NMR spectrum of Tris(2,3,6-triCl)P(4-CH_2Cl)PP.

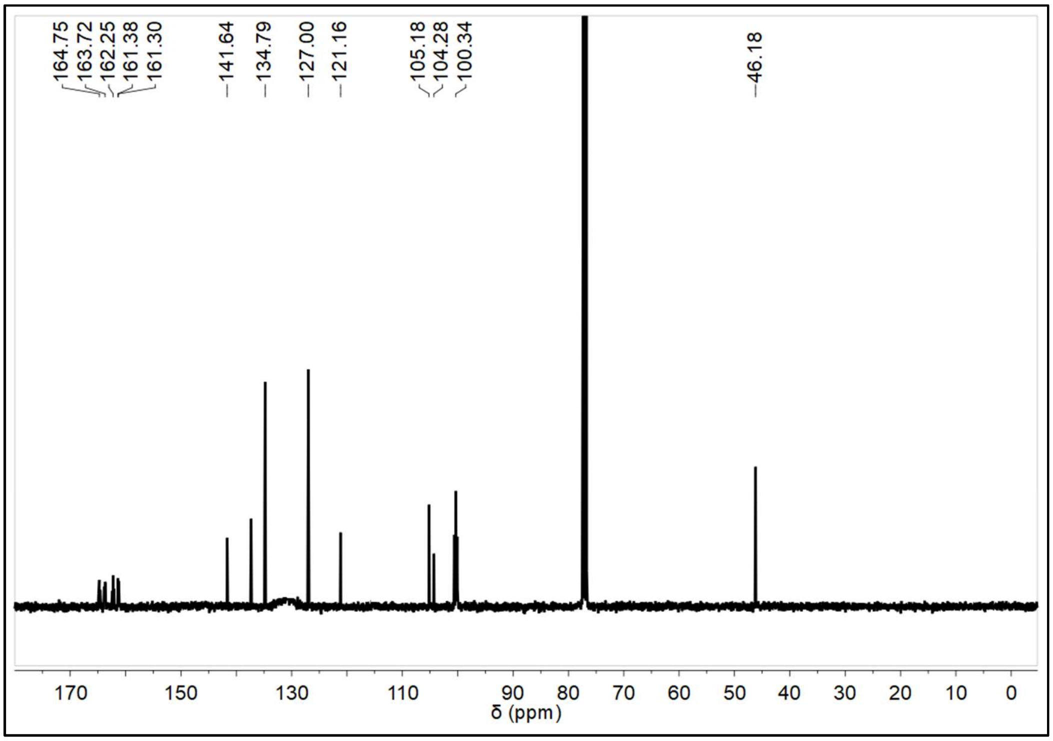

Figure S31. ^{13}C NMR spectrum of Tris(2,4,6-triF)P(4-CH_2Cl)PP.

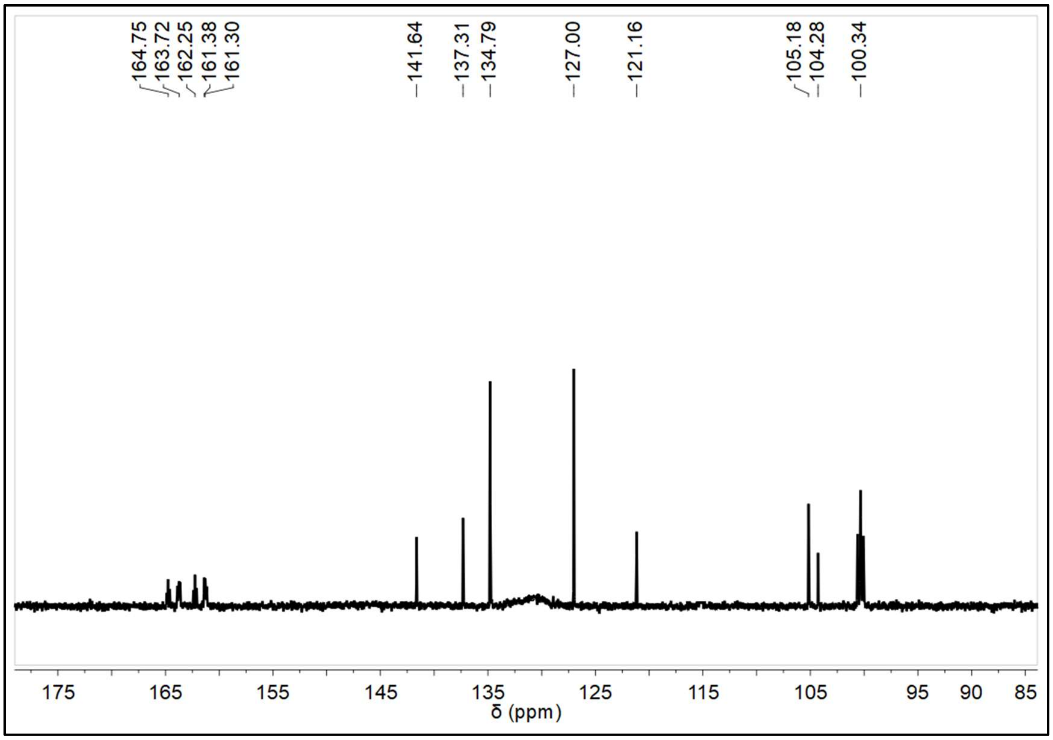

Figure S32. Locally magnified ^{13}C NMR spectrum of Tris(2,4,6-triF)P(4-CH_2Cl)PP.

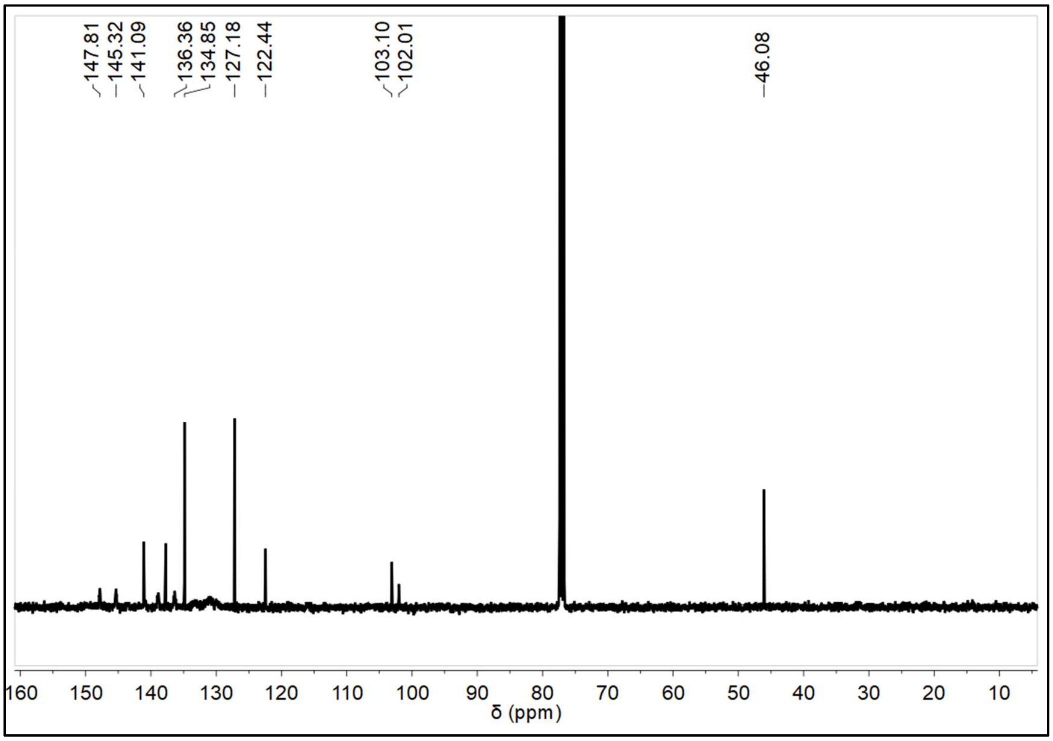

Figure S33. ^{13}C NMR spectrum of Tris(perF)P(4-CH_2Cl)PP.

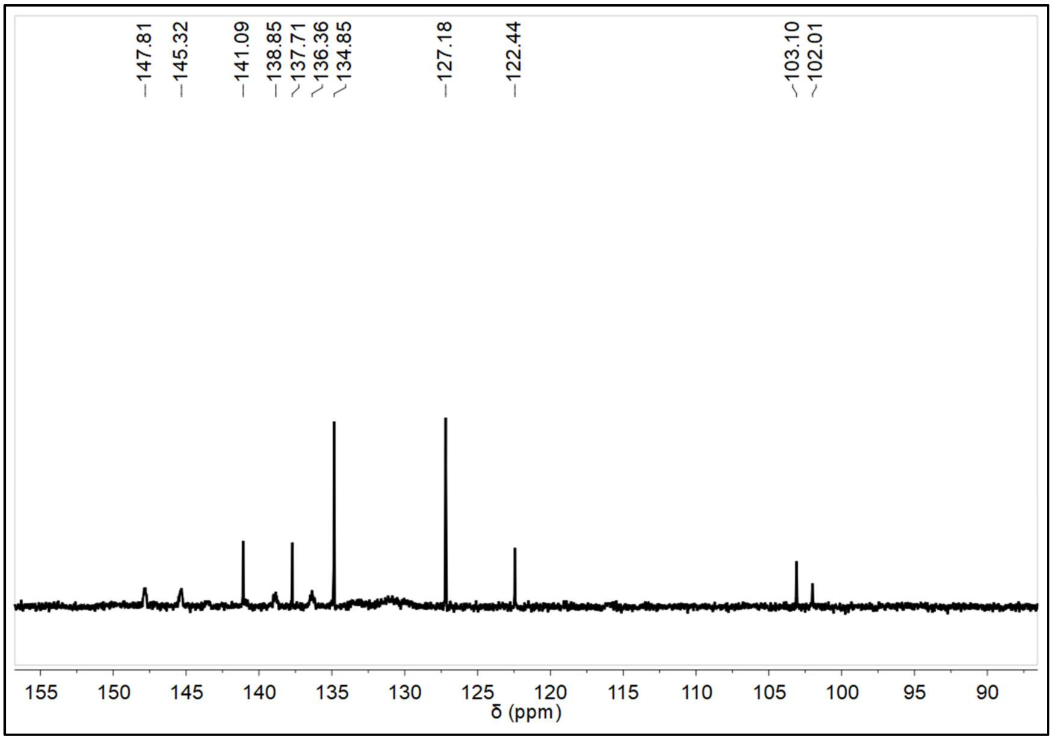

Figure S34. Locally magnified ^{13}C NMR spectrum of Tris(perF)P(4-CH_2Cl)PP.

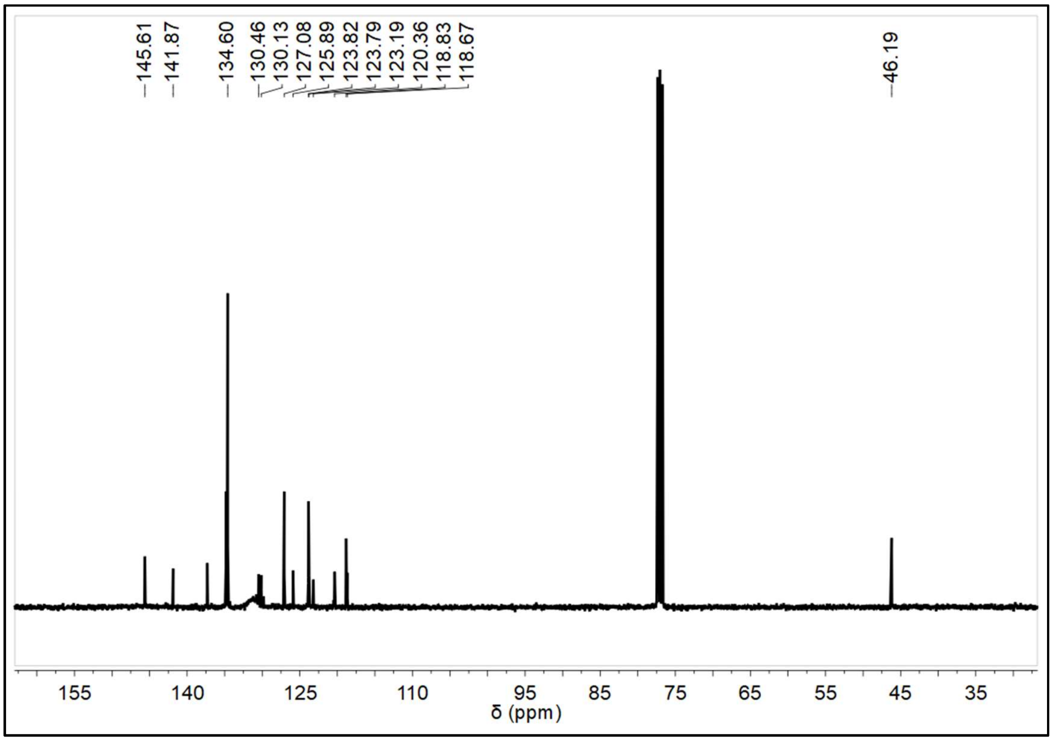

Figure S35. ^{13}C NMR spectrum of Tris(4-CF_3)P(4-CH_2Cl)PP.

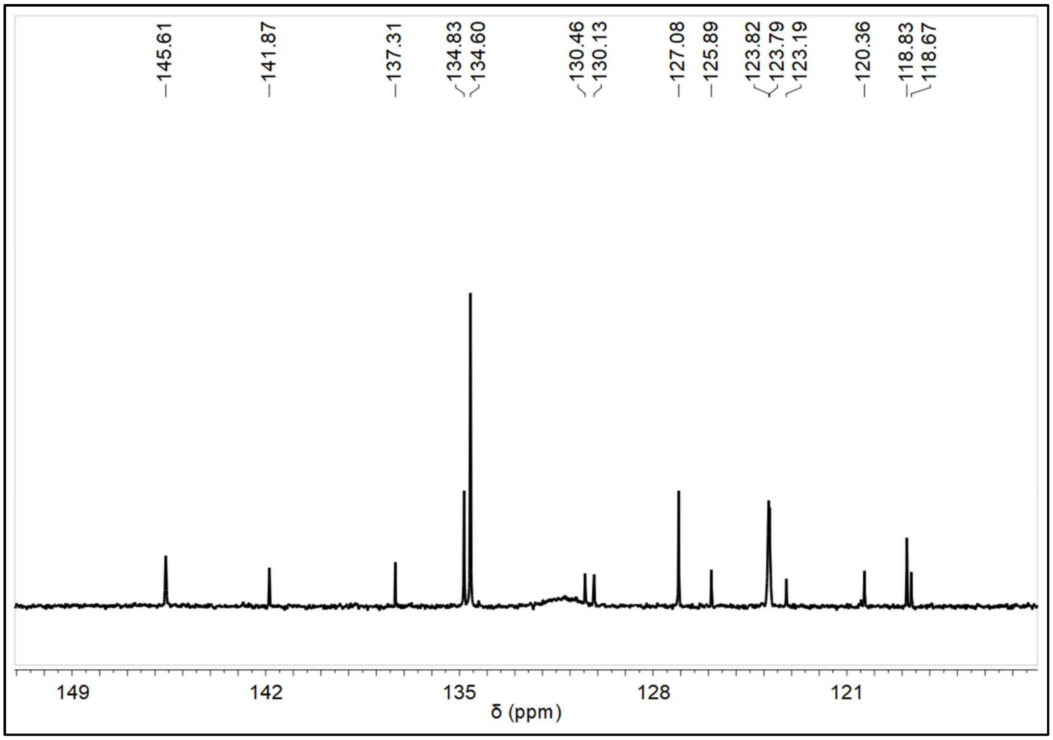

Figure S36. Locally magnified ^{13}C NMR spectrum of Tris(4-CF_3)P(4-CH_2Cl)PP.

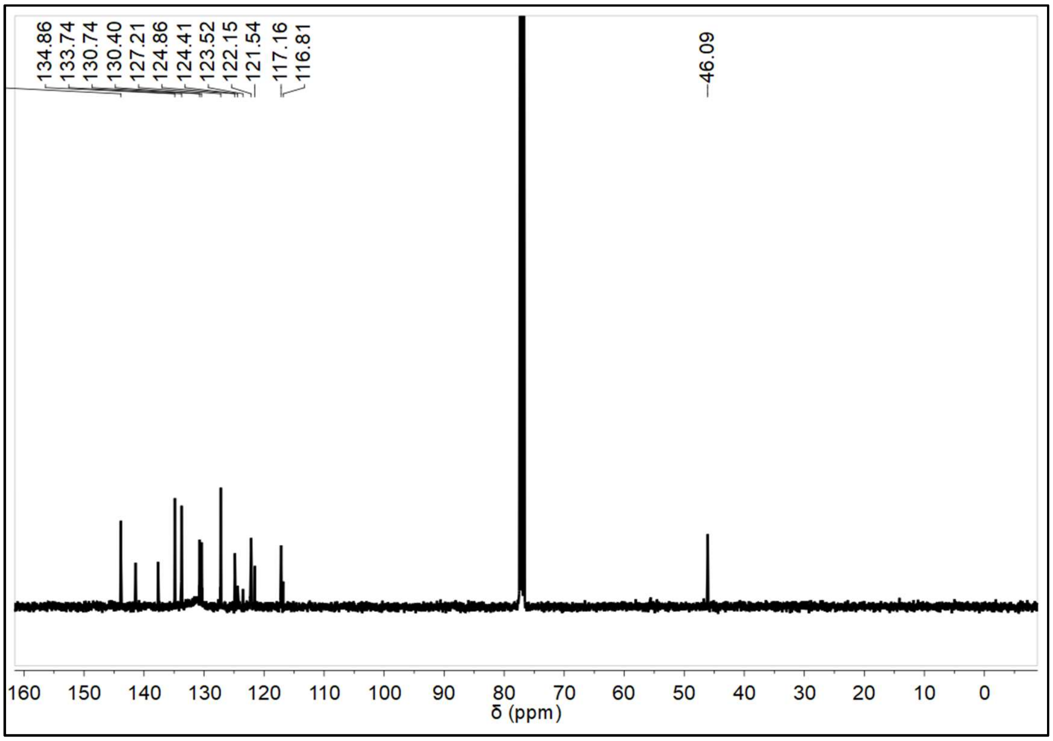

Figure S37. ^{13}C NMR spectrum of Tris(3,5-diCF$_3$)P(4-CH$_2$Cl)PP.

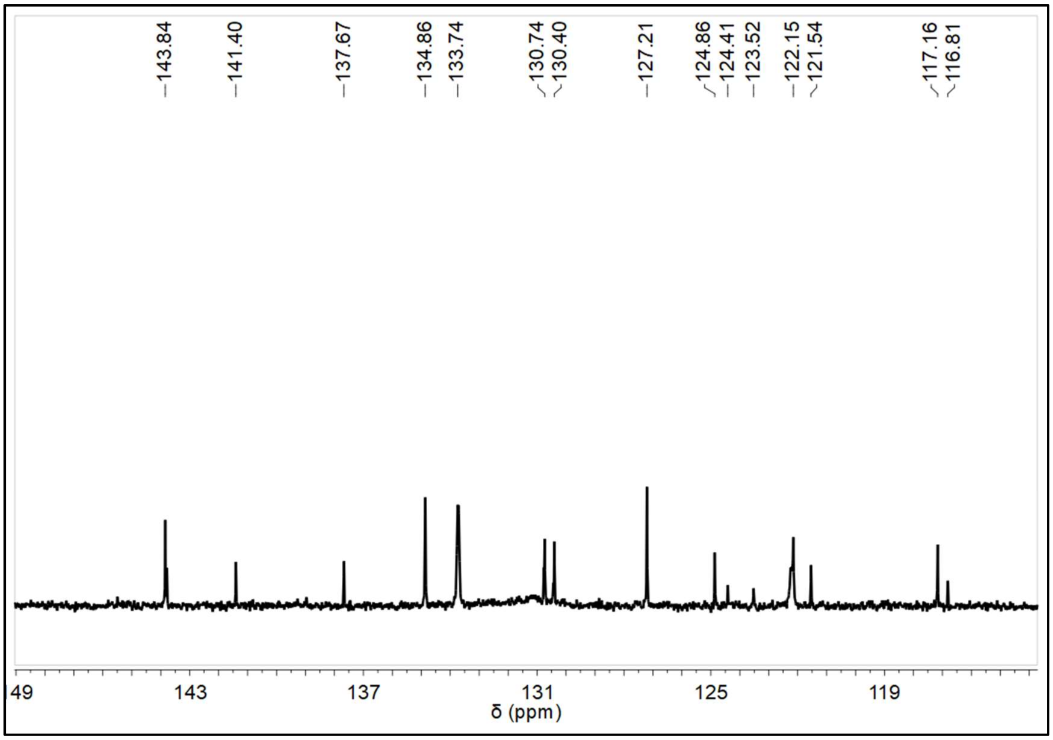

Figure S38. Locally magnified ^{13}C NMR spectrum of Tris(3,5-diCF$_3$)P(4-CH$_2$Cl)PP.

8. ESI-MS spectra of porphyrins and metalloporphyrins

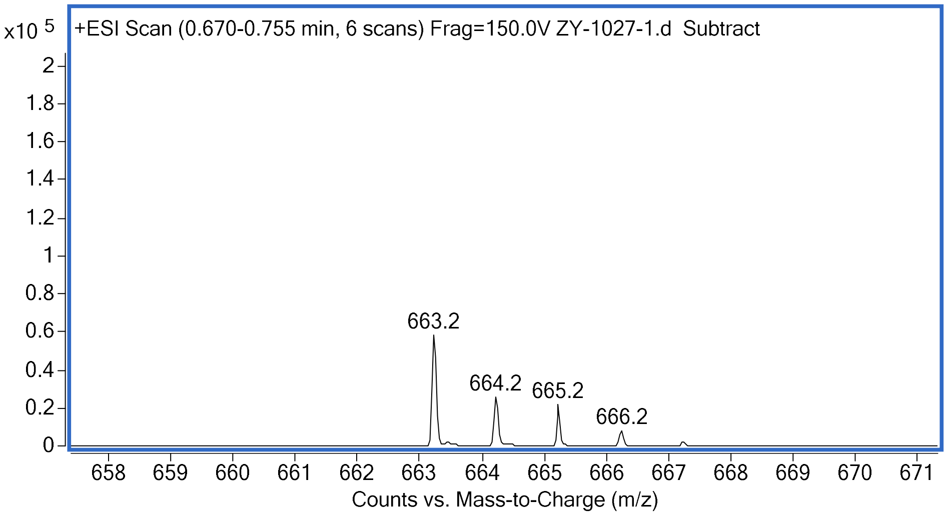

Figure S39. ESI-MS spectrum of TrisP(4-CH_2Cl)PP.

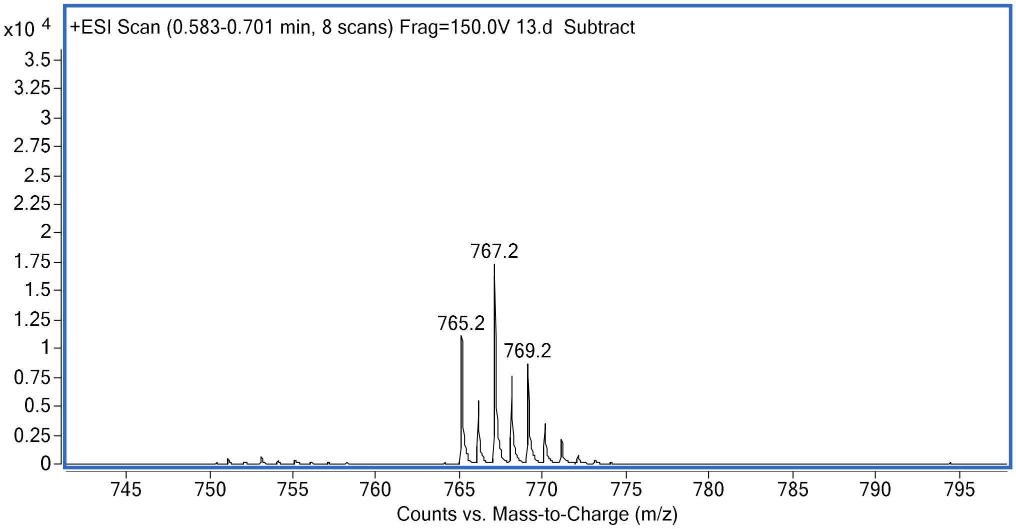

Figure S40. ESI-MS spectrum of Tris(4-Cl)P(4-CH_2Cl)PP.

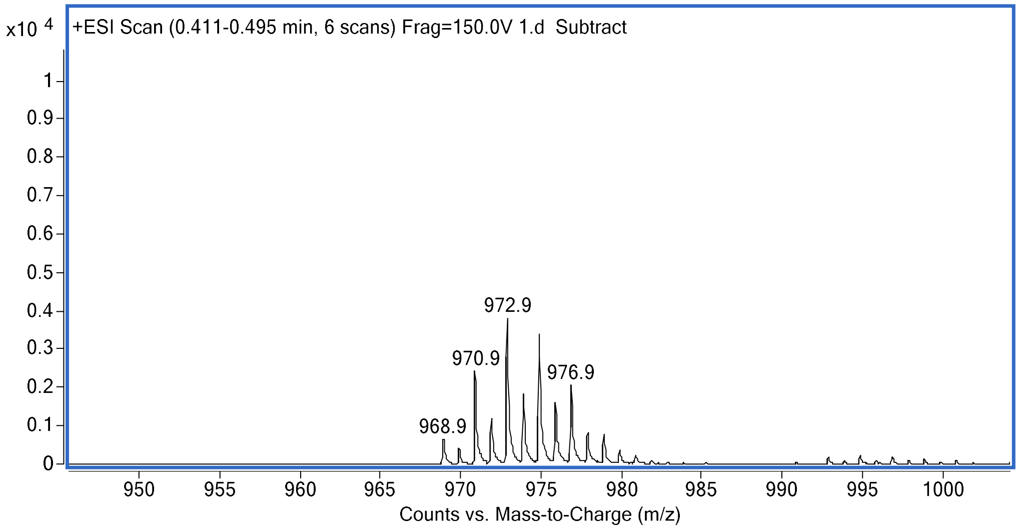

Figure S41. ESI-MS spectrum of Tris(2,3,6-triCl)P(4-CH_2Cl)PP.

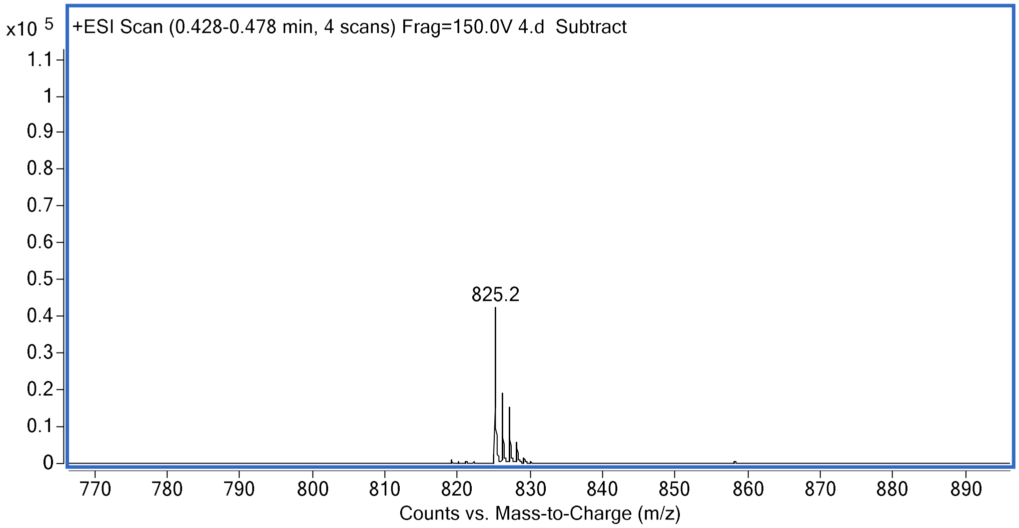

Figure S42. ESI-MS spectrum of Tris(2,4,6-triF)P(4-CH_2Cl)PP.

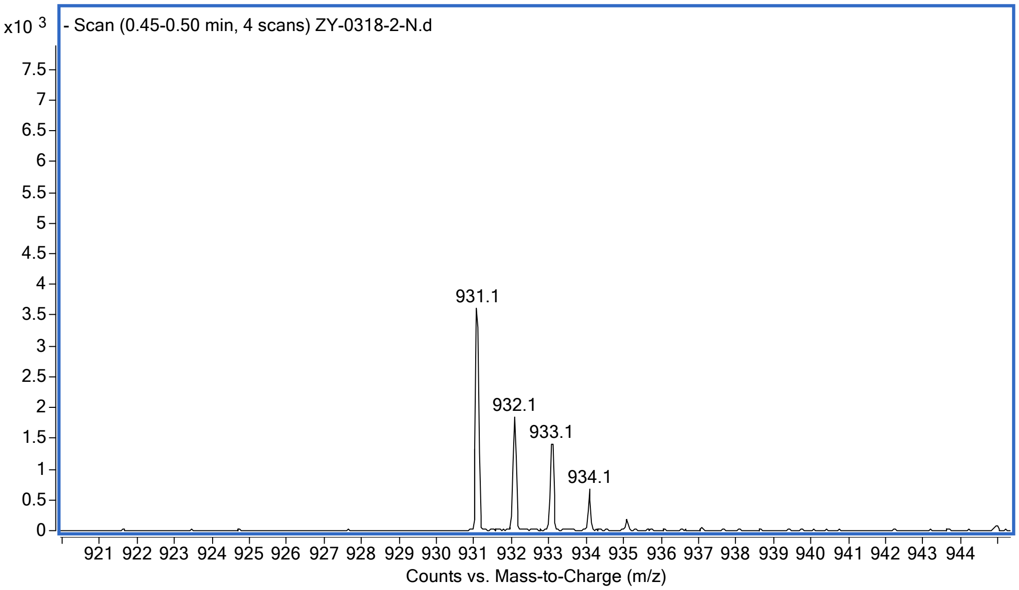

Figure S43. ESI-MS spectrum of Tris(perF)P(4-CH_2Cl)PP.

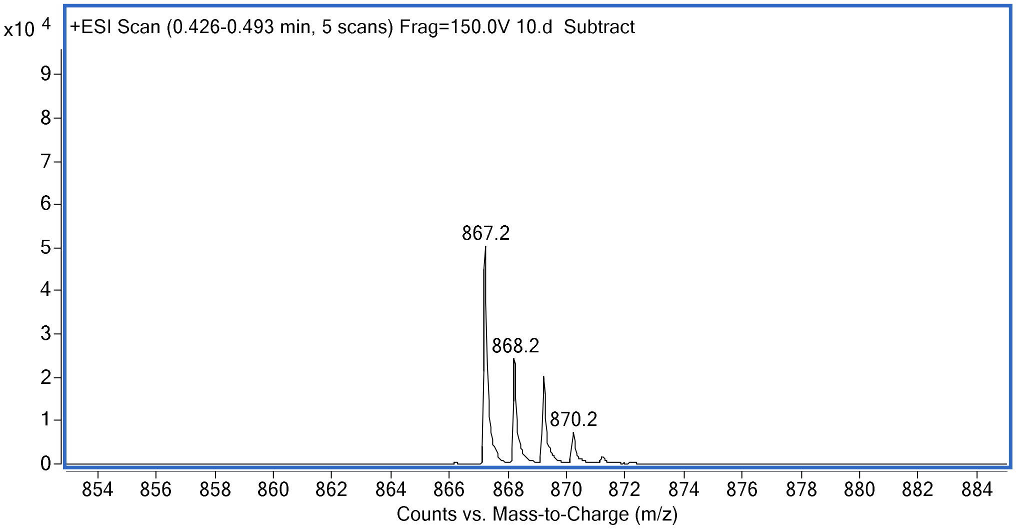

Figure S44. ESI-MS spectrum of Tris(4-CF_3)P(4-CH_2Cl)PP.

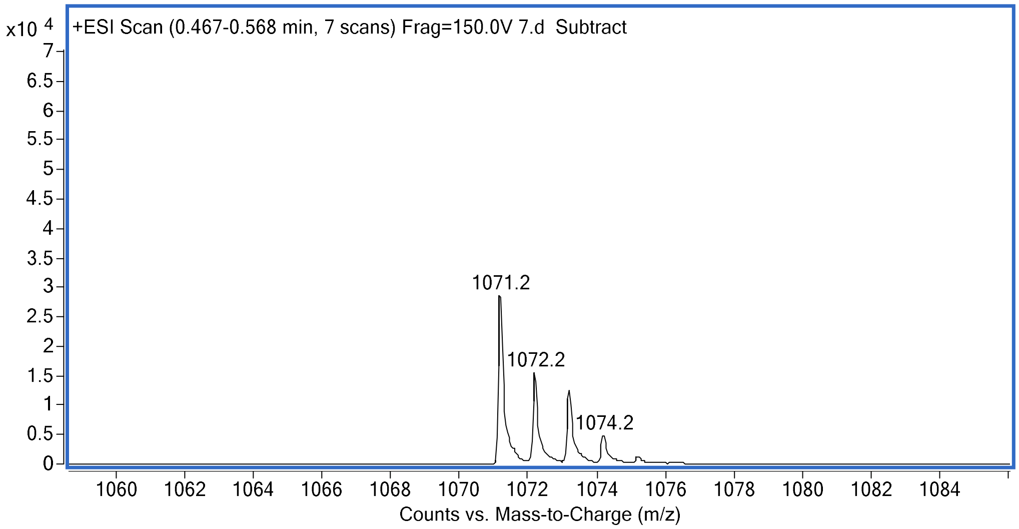

Figure S45. ESI-MS spectrum of Tris(3,5-diCF_3)P(4-CH_2Cl)PP.

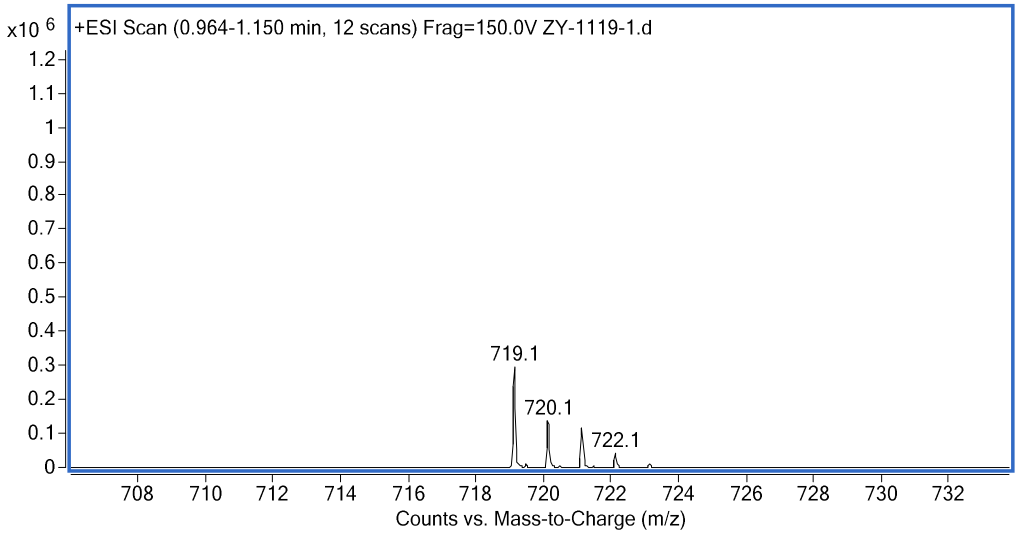

Figure S46. ESI-MS spectrum of TrisP(4-CH_2Cl)PPCo.

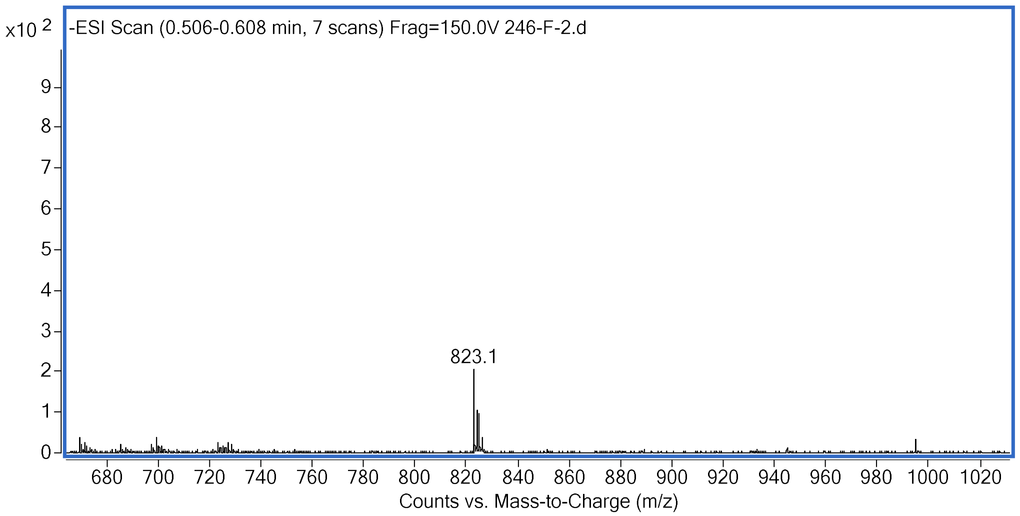

Figure S47. ESI-MS spectrum of Tris(4-Cl)P(4-CH_2Cl)PPCo.

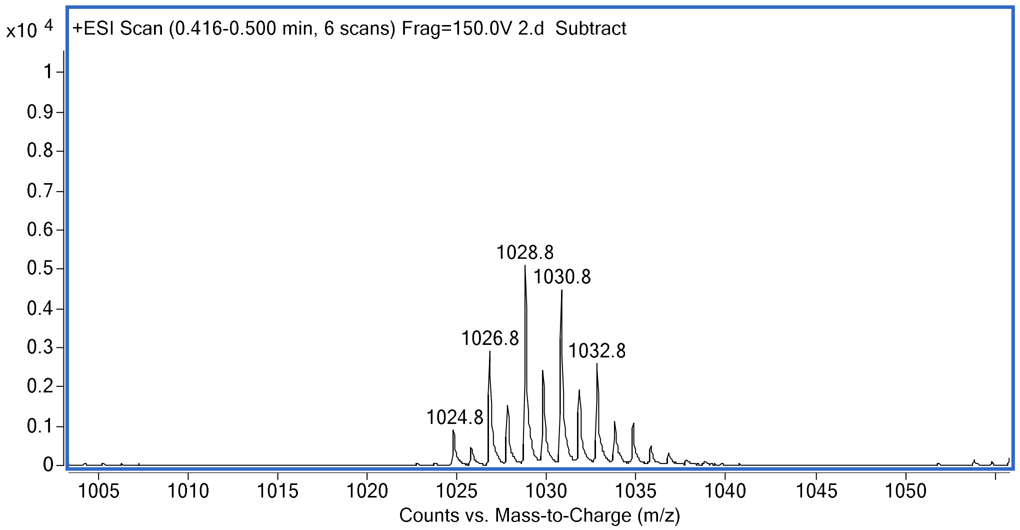

Figure S48. ESI-MS spectrum of Tris(2,3,6-triCl)P(4-CH_2Cl)PPCo.

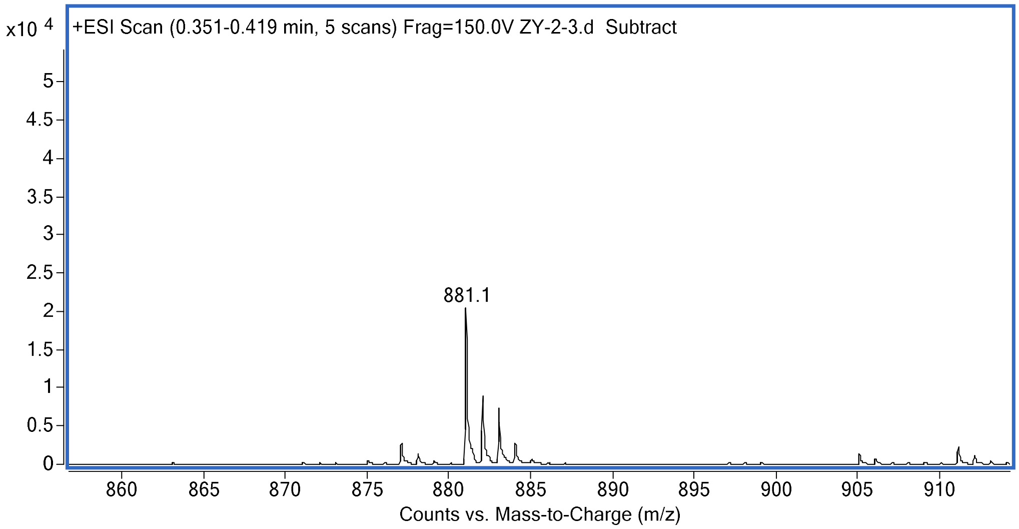

Figure S49. ESI-MS spectrum of Tris(2,4,6-triF)P(4-CH_2Cl)PPCo.

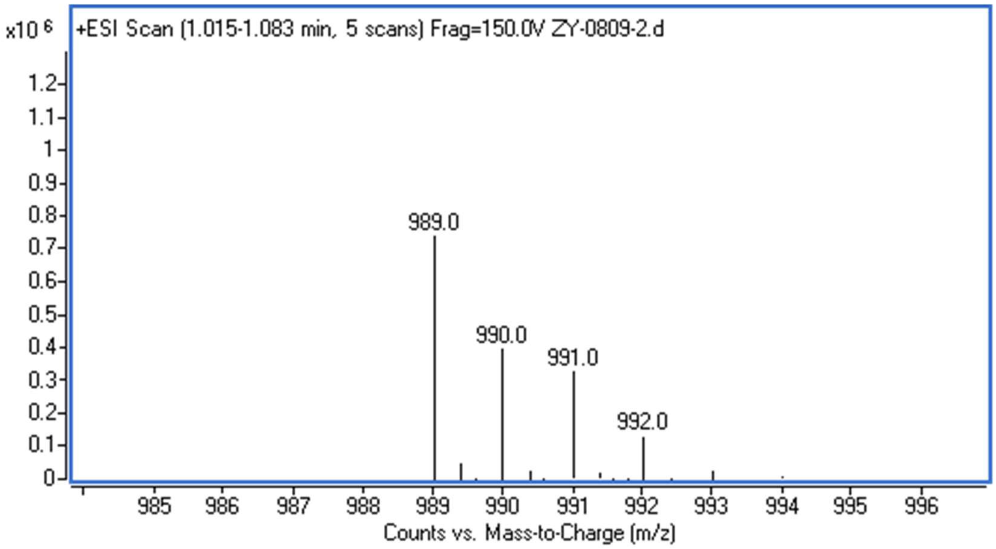

Figure S50. ESI-MS spectrum of Tris(perF)P(4-CH_2Cl)PPCo.

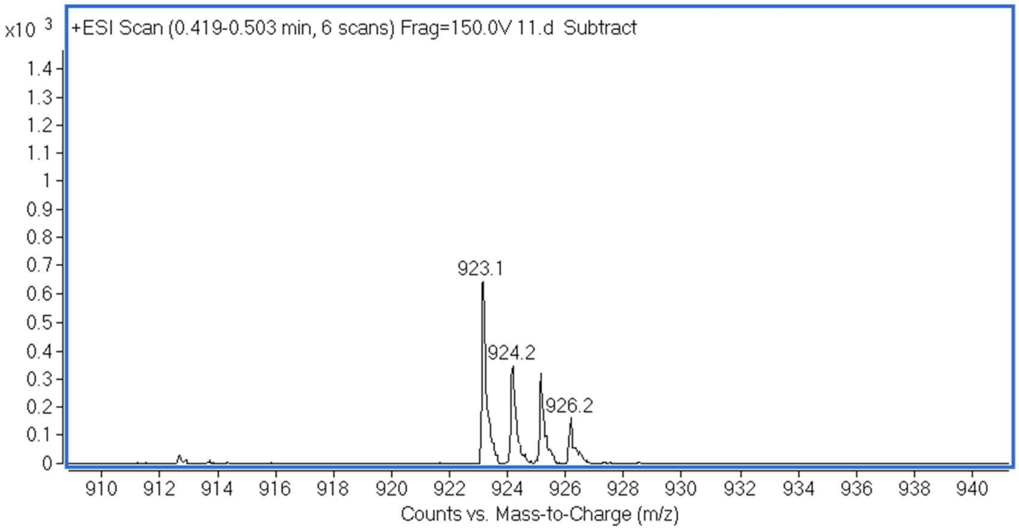

Figure S51. ESI-MS spectrum of Tris(4-CF_3)P(4-CH_2Cl)PPCo.

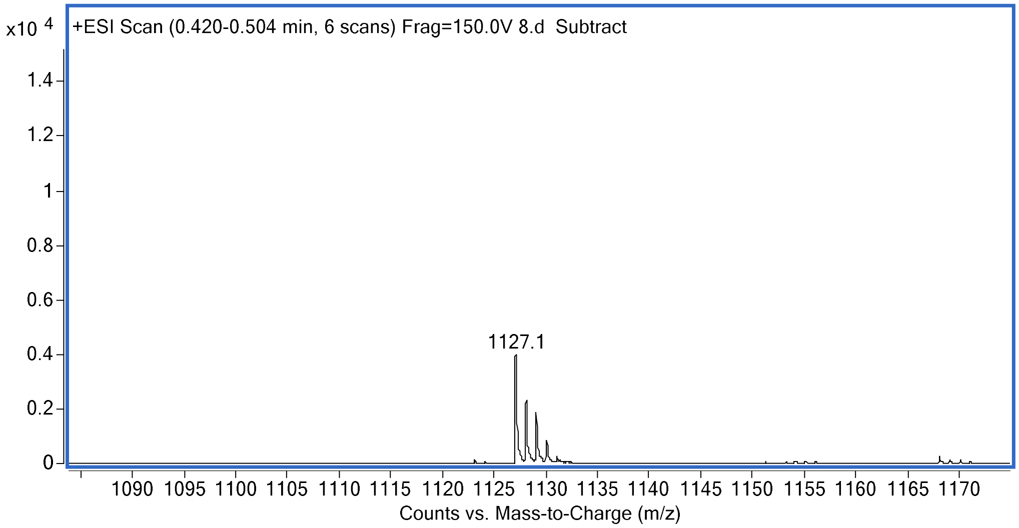

Figure S52. ESI-MS spectrum of Tris(3,5-diCF_3)P(4-CH_2Cl)PPCo.

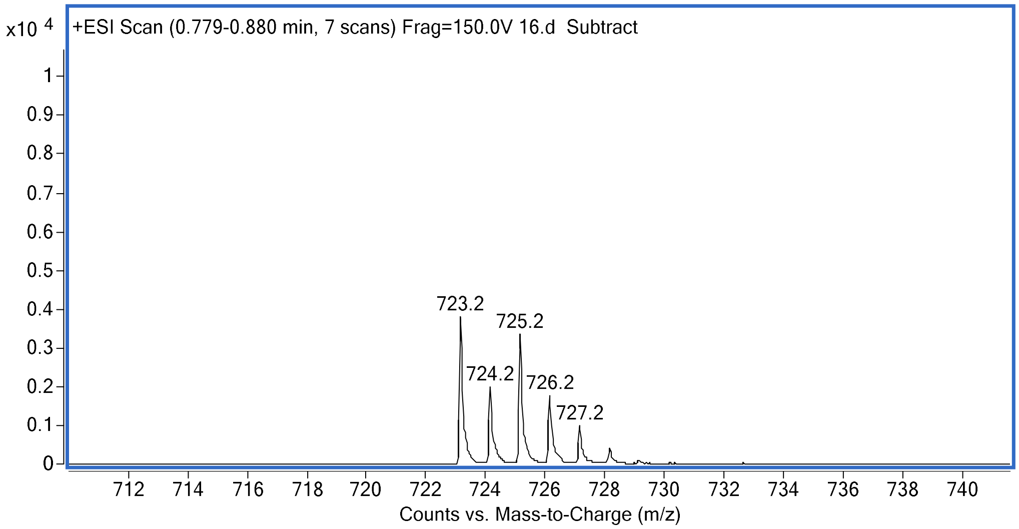

Figure S53. ESI-MS spectrum of TrisP(4-CH_2Cl)PPCu.

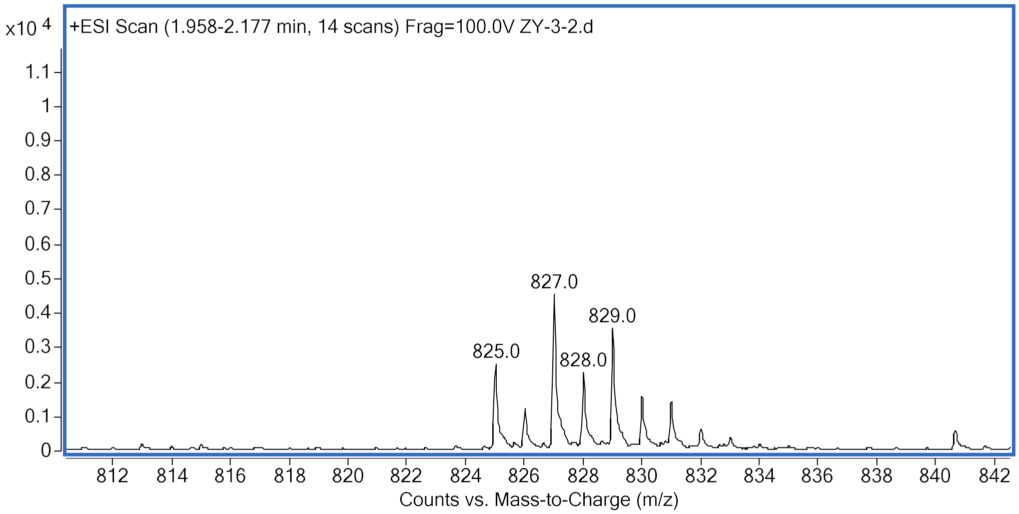

Figure S54. ESI-MS spectrum of Tris(4-Cl)P(4-CH_2Cl)PPCu.

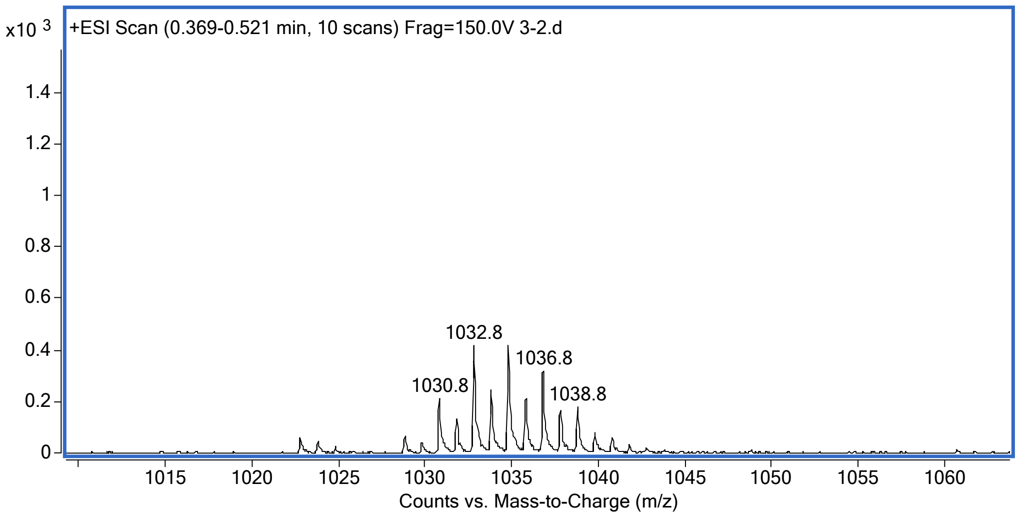

Figure S55. ESI-MS spectrum of Tris(2,3,6-triCl)P(4-CH_2Cl)PPCu.

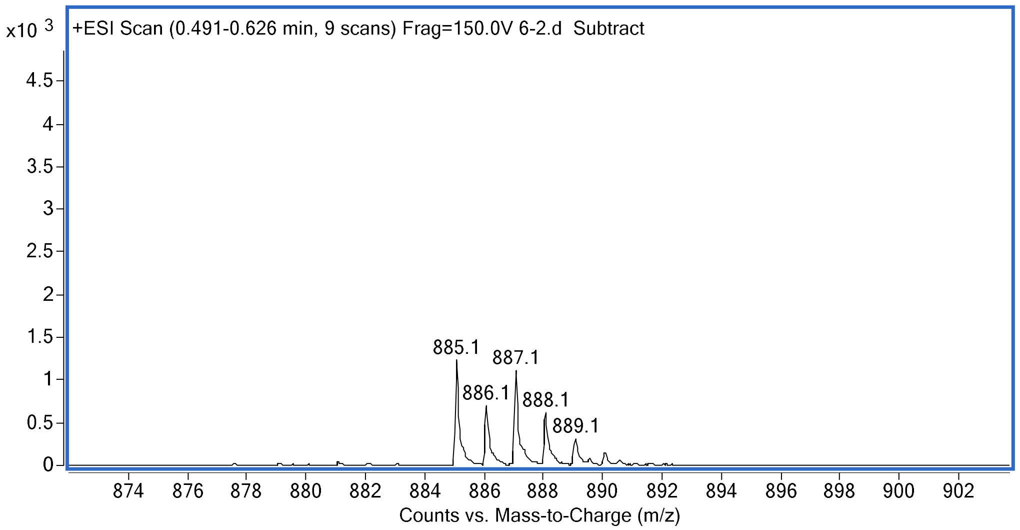

Figure S56. ESI-MS spectrum of Tris(2,4,6-triF)P(4-CH_2Cl)PPCu.

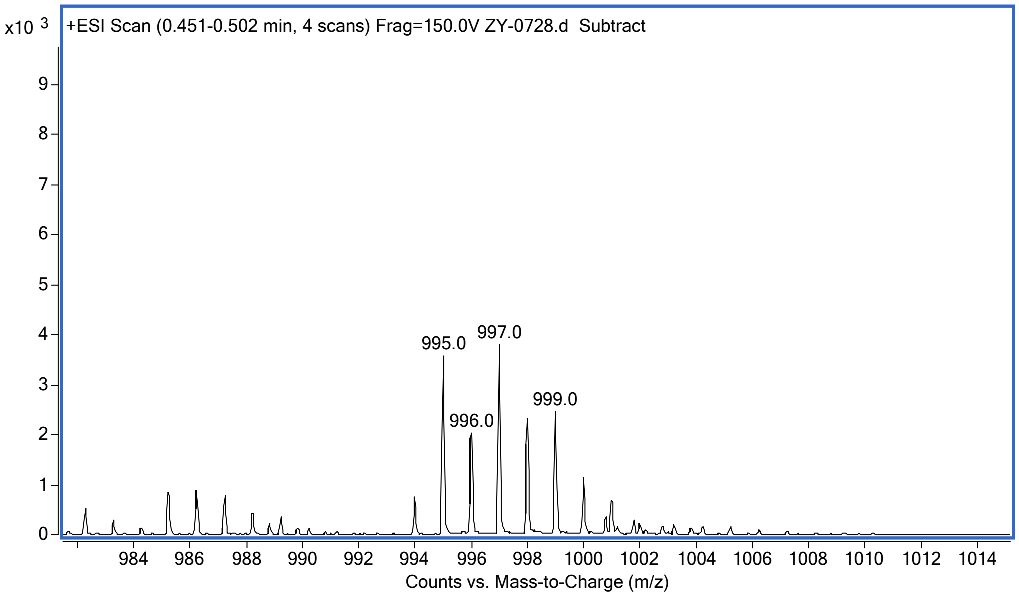

Figure S57. ESI-MS spectrum of Tris(perF)P(4-CH_2Cl)PPCu.

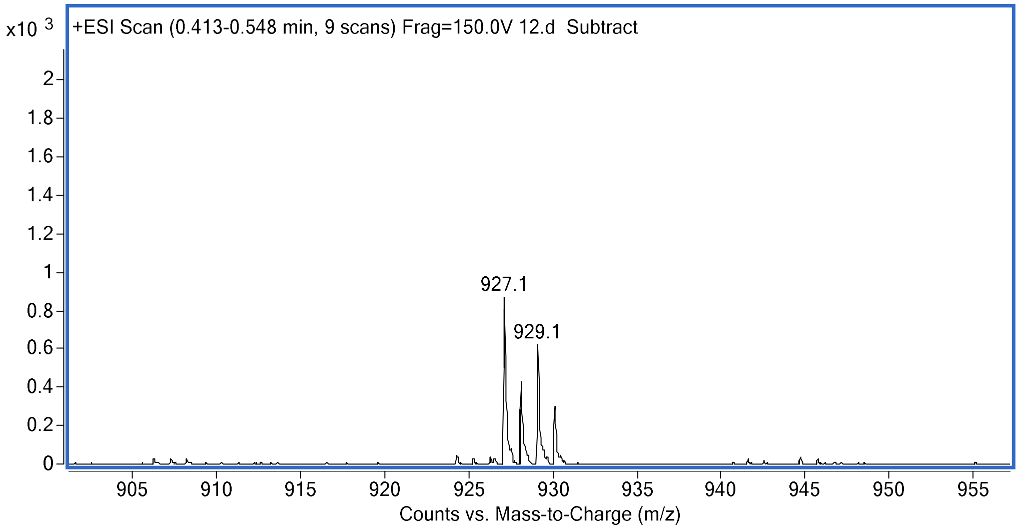

Figure S58. ESI-MS spectrum of Tris(4-CF_3)P(4-CH_2Cl)PPCu.

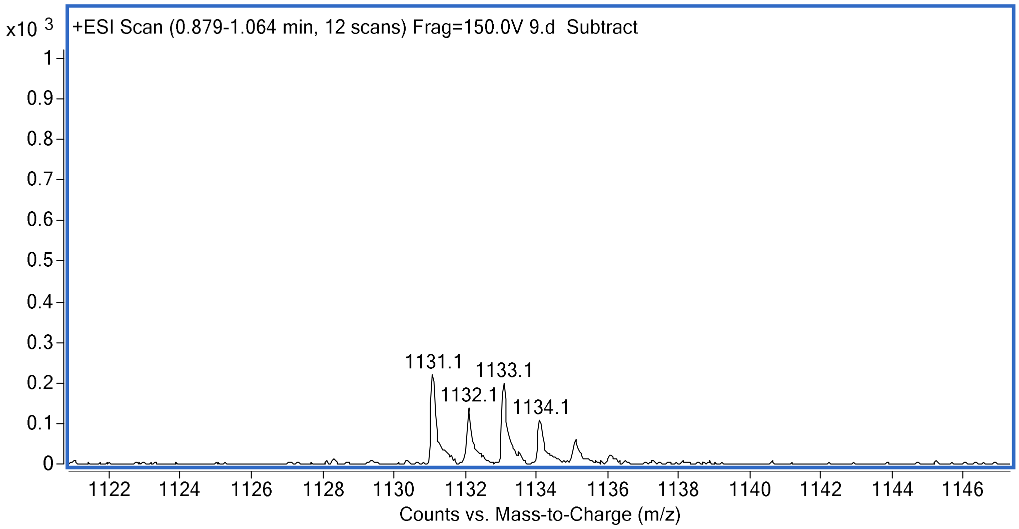

Figure S59. ESI-MS spectrum of Tris(3,5-diCF_3)P(4-CH_2Cl)PPCu.

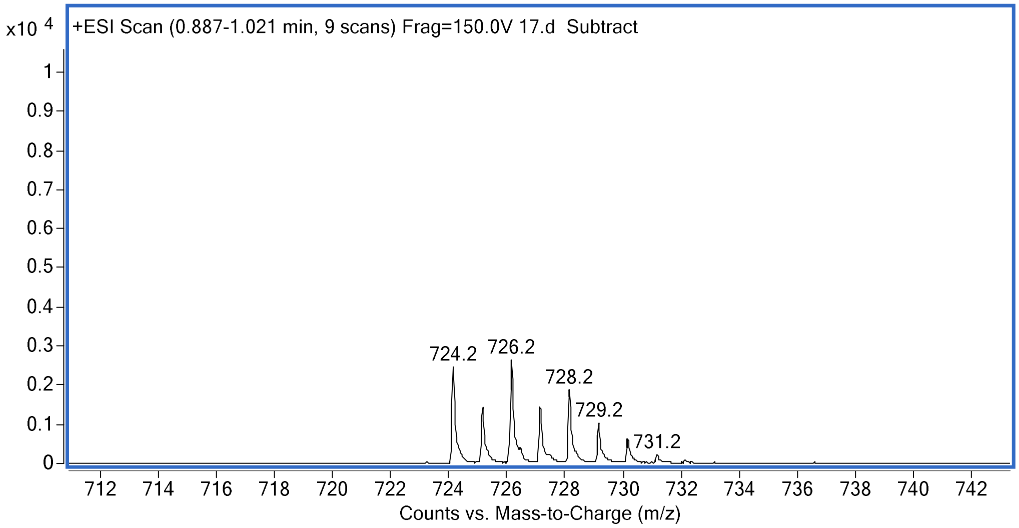

Figure S60. ESI-MS spectrum of TrisP(4-CH_2Cl)PPZn.

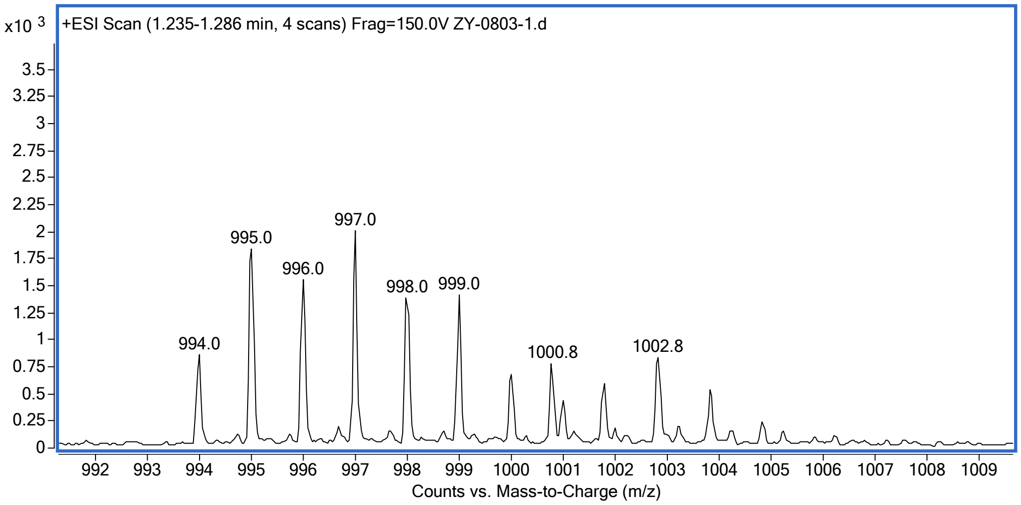

Figure S61. ESI-MS spectrum of Tris(perF)P(4-CH_2Cl)PPZn.
